# CRISPR-Cas9 screening reveals G2E3 as a novel ubiquitin-linked factor controlling autophagosome-lysosome fusion and cancer cell progression

**DOI:** 10.1038/s41420-025-02717-0

**Published:** 2025-10-09

**Authors:** Yumei Gong, Marc Leon, Huaqing Mo, Premkamol Pengpaeng, Hai Yang, Yanxi Lu, Zhiqiang Yin, Alan Benard, Yong Zhou, Robert Grützmann, Christian Pilarsky

**Affiliations:** 1 Department of Surgery, Universitätsklinikum Erlangen, Friedrich-Alexander-Universität, Erlangen-Nürnberg (FAU), Erlangen, Germany; 2 Department of Cardiothoracic Surgery, Stanford University School of Medicine, Stanford, CA USA; 3 Department of Surgery, Juraklinik Scheßlitz Oberend 29, Scheßlitz, Germany; 4 Department of Cardiovascular Surgery, The Second Affiliated Hospital, Zhejiang University School of Medicine, Hangzhou, China

**Keywords:** Preclinical research, Cell division

## Abstract

Autophagy is a tightly regulated process essential for cellular homeostasis, with ubiquitination playing a crucial role in its regulation. However, the specific ubiquitin related factors involved in autophagic flux remain largely unexplored. Identifying these regulators is essential for advancing the mechanistic understanding of autophagy and its broader implications in cellular function. This study aimed to identify novel ubiquitination-associated regulators of autophagy. To achieve this, we conducted a CRISPR-Cas9 loss-of-function screen targeting 660 ubiquitination-related genes in pancreatic cancer cells expressing the mCherry-GFP-LC3 autophagy flux reporter system. Among the top candidates, we identified G2E3, a G2/M-phase-specific E3 ubiquitin ligase, as a previously unrecognized autophagy regulator. Subsequent functional analyses revealed that G2E3 knock out led to a significant accumulation of LC3B-II and GABARAPs, indicative of impaired autophagic flux. Further confocal imaging demonstrated that the co-localization of LC3B with LAMP1-positive lysosomes was significantly reduced in G2E3 knock out cells, suggesting defective autophagosome-lysosome fusion. Mechanistically, G2E3 directly interacts with GABARAP and GABARAPL1, but not LC3B, positioning it as a key regulator of late-stage autophagy. Additionally, G2E3 knock out cells exhibited reduction in migration and invasion capability, suggesting its role in cancer progression. These findings establish G2E3 as a novel ubiquitin-related regulator of autophagy, specifically facilitating autophagosome-lysosome fusion via a GABARAPs-dependent mechanism. This study reveals a previously unrecognized role of G2E3 in late-stage autophagy and suggests that targeting G2E3 could provide a potential therapeutic approach for modulating autophagy-dependent cellular processes, including cancer progression.

## Introduction

Pancreatic ductal adenocarcinoma (PDAC) exhibits elevated autophagy, which supports tumor growth, promotes immune evasion, and enhances therapeutic resistance [1]. Although autophagy inhibition has been explored as a potential treatment strategy, existing approaches have shown limited clinical benefits, indicating a need for more precise regulatory targets [2, 3]. Autophagy is a complex, multi-step process, with the fusion of autophagosomes and lysosomes being a critical step for effective degradation. This process is primarily regulated by the ATG8 protein family, where LC3 subfamily proteins contribute to autophagosome membrane elongation, while the GABARAP subfamily(GABARAPs) plays a crucial role in autophagosome-lysosome fusion [4, 5]. Current studies highlight the involvement of GABARAPs in recruiting PLEKHM1, which facilitate fusion by interacting with RAB7 and the HOPS complex. Autophagy was shown to be regulated by ubiquitination at multiple steps [6]. For example, UBA6 and BIRC6 function as E1 and E2/3 enzymes, respectively, to mono-ubiquitize LC3B, negatively regulating autophagy [7]; USP32 deubiquitinase reverses a ubiquitin signal on LAMTOR1, impacting mTORC1 and the initination of autophagy [8]. However, the multi-functional and context-dependent nature of known autophagy regulators presents challenges for their clinical application [2]. Therefore, it is necessary to uncover additional selective autophagy regulators to fully elucidate the intricate autophagy network and its therapeutic potential.

To identify novel autophagy regulators, we conducted a CRISPR-Cas9 loss-of-function screen targeting 660 ubiquitination-related genes in pancreatic cancer cells and identified G2E3, a G2/M-phase-specific E3 ubiquitin ligase, as a potential autophagy regulator. To test this, we employed gene knockout models, fluorescence-based autophagy flux assays, confocal microscopy, and co-immunoprecipitation experiments to elucidate the function of G2E3 in autophagy regulation. This study aims to define the molecular mechanism of G2E3 in autophagy and explore its potential as a therapeutic target for PDAC.

## Results

### CRISPR-Cas9 loss-of-function screen identifies novel autophagy regulators inpancreatic cancer cells

**Fig. 1:**
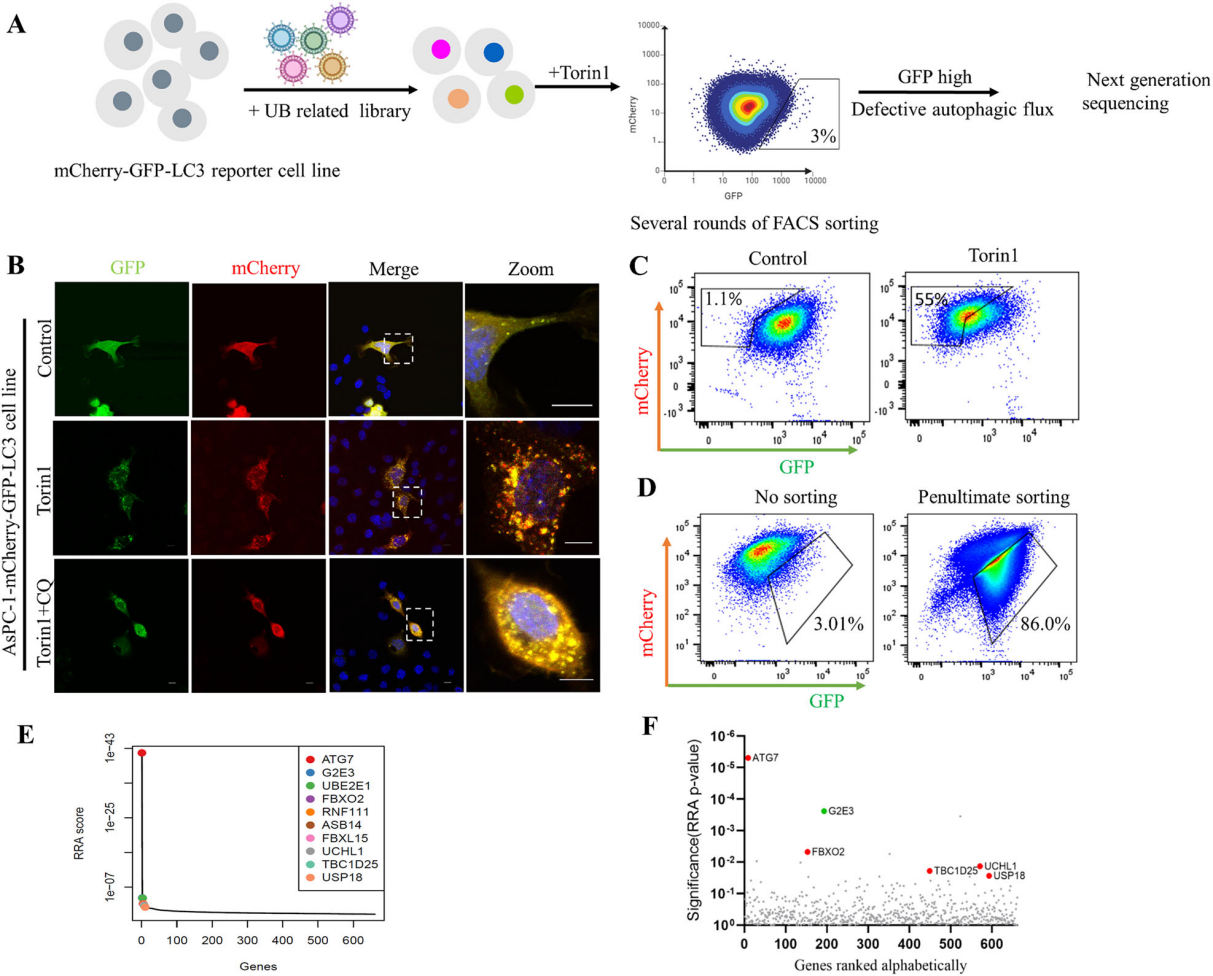
CRISPR-Cas9 KO screen for the identification of novel autophagy genes. **A** Schematic representation of the FACS based CRISPR-Cas9 screen. AsPC-1 cells stably expressing mCherry-GFP-LC3 were mutated with the lentiviral ubiquitination-related library. After selected with puromycin, cells were treated with Torin1 for 12 h and high GFP signal were sorted and propagated; after five rounds of sorting, genomic DNA was isolated and analyzed with next-generation sequencing. **B** Confocal microscopy pictures of AsPC-1 cells stably expressing GFP-mCherry-LC3. Cells were incubated in the absence (control) or treated with Torin1 (concentration of Torin1, 100 nM) for 12 h with or without CQ (concentration of CQ, 2 μM).Scale bar: 10 μm. **C** AsPC-1 cells expressing mCherry-GFP-LC3 that sensitive to Torin1 were incubated without (control) or with 100 nM Torin1 for 12 h, the GFP/mCherry fluorescence was measured by FACS. **D** FACS analysis of first-sorted cell populations and penultimate-sorted cell populations of cells transduced with lentiviral libraries. The percentages of cell population in the boxed areas are indicated. **E** Comparison of robust rank aggregation (RRA) score distribution indicated that ATG7 and G2E3 were the top hits. **F** Ranking of genes based on RRA p-value analyzed with the MAGeCK. The top 10 genes known to be involved in autophagy are labeled in red; G2E3 is labeled in green.

To uncover potential autophagy regulators, we applied a pooled CRISPR-Cas9 knock- out (KO) screen to identify novel regulators of autophagy induced by Torin1. Here, we generated human pancreatic cancer reporter cell line stably expressing mCherry-GFP-LC3 to measure autophagic flux. This dual-fluorescent reporter system exploits the pH sensitivity of GFP and stability of mCherry in acidic environments. When autophagosomes fuse with lysosomes, the acidic pH quenches GFP fluorescence while mCherry remains stable. By monitoring the ratio of mCherry to GFP fluorescence, we can quantitatively assess autophagic activity in these cells [7, 9]. Under basal conditions, autophagic cells exhibit reduced GFP signal, whereas cells bearing defective autophagy retain elevated signal of GFP. Using FACS (fluorescence-activated cell sorting), we isolated AsPC-1 cells with a low GFP: mCherry ratio following Torin1 treatment. After two rounds of sorting, Torin1-sensitive AsPC-1 cell population were obtained (Supplementary Fig. 1A). Compared to the unsorted cells (Supplementary Fig. 1B), these Torin1-sensitive AsPC-1 reporter cells exhibited more pronounced reduction in the FACS-based GFP ratio upon Torin1 exposure, confirming their enhanced responsiveness to autophagic induction (Fig. 1C).

Upon autophagy induction by Torin 1 treatment, AsPC-1 WT cells displayed accumulation of mCherry-only puncta (i.e., autolysosomes) and a decreased ratio of GFP: mCherry in FACS analysis (Fig. 1B, C), consistent with the formation of autolysosomes and intact autophagic flux. In the basal state, cells exhibit lower autophagy capacity, explaining why no significant change in the GFP: mCherry ratio was observed by FACS following CQ (chloroquine) treatment (Supplementary Fig. 1C). Notably, lysosomal neutralization with the inhibitor CQ reversed Torin1-induced GFP quenching, leading to an increase in dual-labeled mCherry-GFP puncta (i.e., autophagosomes) (Fig. 1B). This finding indicates inhibited autophagic flux, as CQ blocks lysosomal acidification required for autophagosome-lysosome fusion. Together, these results demonstrate that the AsPC-1-mCherry-GFP-LC3 report cell line exhibits robust responsiveness to both autophagic inhibition and induction.

To identify ubiquitin-related proteins involved in the autophagic machinery, we employed a pooled library containing 11108 sgRNA targeting 660 ubiquitin related protein (E1, E2, E3 ligases, and deubiquitinating enzymes) and 1000 non-targeting sgRNAs to mutagenize the genome of pancreatic cancer cell line AsPC-1. Following transduction of the ubiquitin library into reporter cell lines, autophagy was induced with Torin1, and the top 3% of cells with increased GFP: mCherry ratios (indicative of autophagic defects) were collected (Supplementary Fig. 1D). These cells were sequentially expanded and sorted for an additional four rounds, resulting in an enrichment of high GFP: mCherry ratio cells from 3% to around 90% (Fig. 1D). Genomic DNA from these autophagy-defective cells was extracted and subjected to next-generation sequencing. Differential gene enrichment between the sorted high-GFP group and unsorted control was calculated using the MAGeCK-VISPR software. Among the top ten genes, known autophagy regulators such as ATG7, FBXO2, TBC1D25, UCHL1, USP18 were identified (Fig. 1E, F), demonstrating the reliability of our screen method. G2E3, a novel hit with the highest scores in this screen, has not been previously implicated in autophagy regulation. Therefore, the role of G2E3 in autophagy regulation prompts us to do further investigation.

### G2E3 regulates autophagic flux

**Fig. 2:**
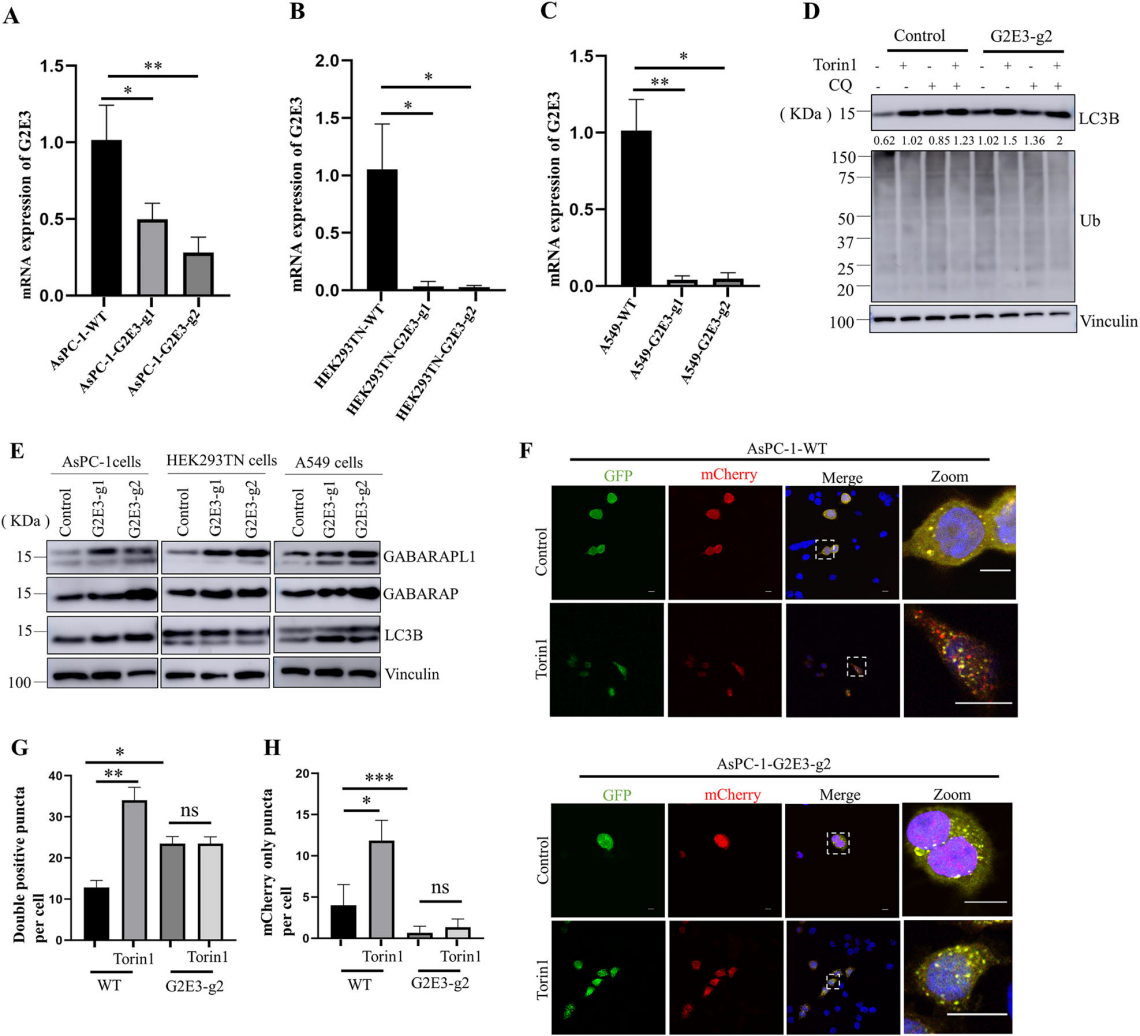
G2E3 is required for autophagy flux. qPCR results showing that G2E3 was knocked out in AsPC-1 (**A**), HEK293TN (**B**) and A549 (**C**) cells. Data were presented as mean ± SEM from three independent experiments.*p < 0.05, **p < 0.01. **D** Protein levels of autophagy markers in wild type and G2E3 KO of AsPC-1 under basal (Control) and Torin1- treated conditions with or without CQ. **E** SDS-PAGE and immunoblotting of lysates from WT or G2E3 KO of AsPC-1, HEK293TN and A549 cells with antibodies to the proteins on the right. **F** Confocal microscopy images of WT and G2E3 KO AsPC-1 cells stably expressing mCherry-GFP-LC3 in different conditions. Scale bar:10 μm. **G**, **H** The number of double positive puncta (i.e., mCherry-GFP–positive puncta) or mCherry only puncta in WT and G2E3 KO AsPC-1 cells treat with or without Torin1 were calculated. Data were presented as mean ± SEM in 10 cells from three independent experiments. n.s. not significant, *p < 0.05, **p < 0.01, ***p < 0.001, according to an unpaired Student’s *t* test.

To investigate the involvement of G2E3 in autophagy, we generated G2E3 KO AsPC-1 cells by using the CRISPR-Cas9 system and the monoclones were isolated. QPCR and DNA-sequencing were used to detect the knockout effects of G2E3 and the clones displayed a one base indel in the G2E3 coding sequence leading to a frameshift mutation (Aspc-1-G2E3-g1 deletion; Aspc-1-G2E3-g2 insertion; Fig. 2A–C) Followed, we checked the protein levels of LC3B and ubiquitin signal in mammalian cell lines.

As expected, Torin1 treatment increased LC3B levels in AsPC-1 WT cells, while CQ treatment inhibited LC3B degradation-particularly the lipidated form LC3B-II. Given that the LC3B antibody exhibits higher affinity for the lower band (with the upper band being undetectable), we quantified the density of the lower band using ImageJ (Fig. 2D). Interestingly, G2E3 KO AsPC-1 cells displayed elevated LC3B-II protein levels compared to parental WT cells, under both basal and Torin1-induced conditions, with no detectable changes in ubiquitin signals (Fig. 2D). We further generated G2E3 KO cell lines in A549 human lung cancer cells and HEK293TN human embryonic kidney cells using the CRISPR-Cas9 system. Similar increases in LC3B-II, GABARAP, and GABARAPL1 levels were observed in G2E3 KO A549 and AsPC-1 cell line. By contrast, G2E3 KO HEK293TN cells displayed only increased GABARAP and GABARAPL1 levels, with no change in LC3B (Fig. 2E). This findings contrast with ATG7 KO in HEK293TN and A549 cells, which led to increased levels of LC3B-I, GABARAPL1-I and P62, but reduced levels of LC3B-II and GABARAPL1-II (Supplementary Fig. 2A–C).

We generated a G2E3 KO AsPC-1 reporter cell line by transducing G2E3 KO monoclonal cells with the lentivirus of mCherry-GFP-LC3. Confocal microscopy analysis revealed that during Torin1-induced autophagy cascade, WT AsPC-1 cells expressing mCherry-GFP-LC3 displayed mCherry-positive but GFP-negative puncta, indicative of intact autophagic flux. In contrast, most of the autophagosomes induced by Torin1 in G2E3 KO ASPC-1 cells expressing the mCherry-GFP-LC3 reporter remained mCherry-GFP double positive (Fig. 2G, H), suggesting that autophagic flux was impaired by G2E3 deletion. Together, these findings demonstrate that G2E3 is involved in the process of autophagy and its deletion inhibits autophagic flux.

### G2E3 interacts and co-localizes with GABARAP and GABARAPL1

**Fig. 3:**
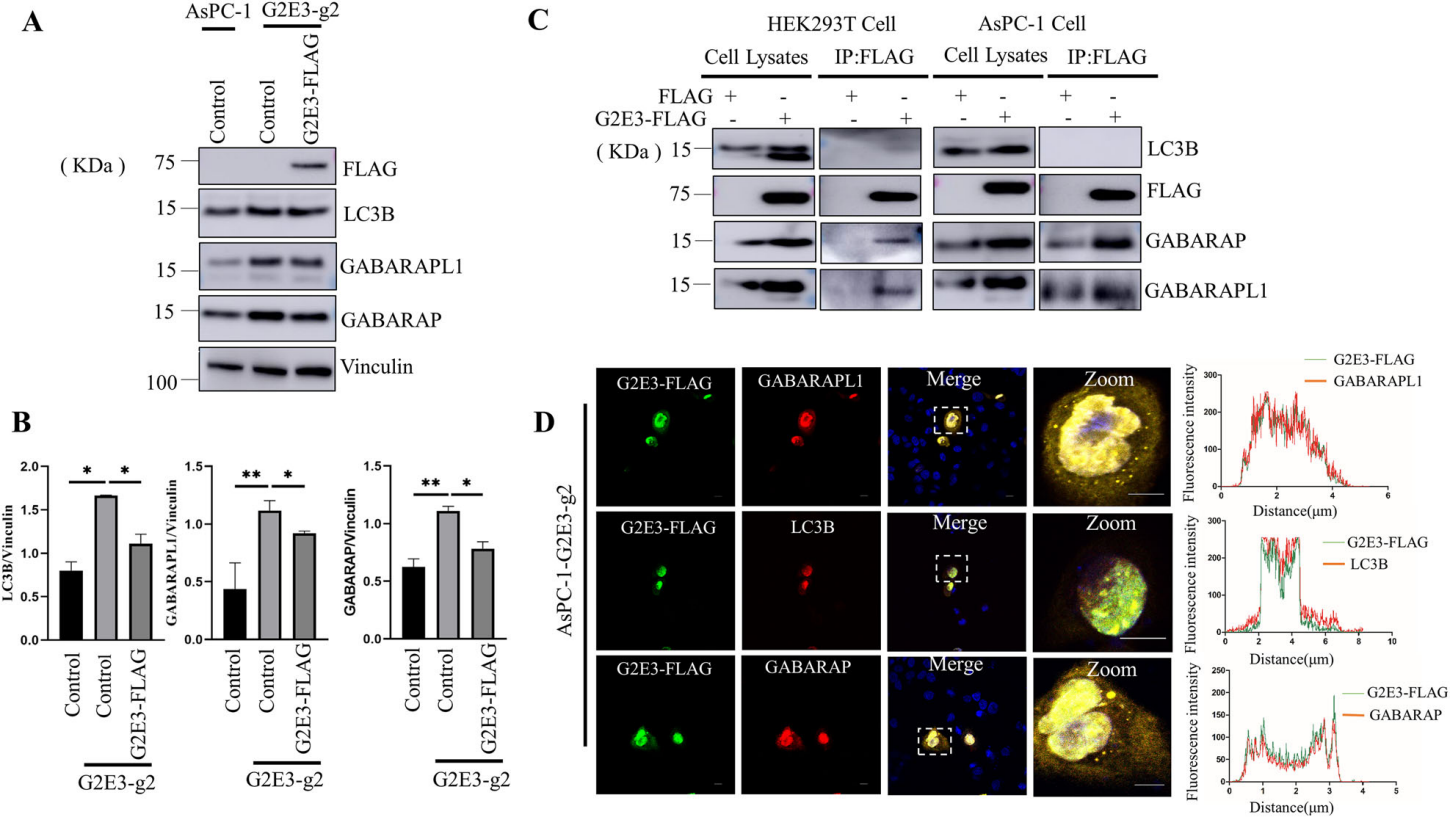
G2E3 interacts specifically with GABARAP and GABARAPL1. **A** WT or G2E3-KO AsPC-1 cells were transfected with control plasmid or plasmids encoding G2E3-FLAG. The cells were then analyzed by SDS-PAGE and immunoblotting with antibodies to the indicate proteins. **B** Quantification of ratio of LC3B-II, GABARAP and GABARAPL1 to Vinculin. The indicated p-values were calculated using means of three independent experiments ±SEM. n.s., not significant, *p < 0.05, **p < 0.01. Significance was calculated with Student’s *t* test. **C** HEK293TN WT and AsPC-1 WT were transfected with plasmids encoding G2E3-FLAG or control plasmid FLAG. After 48 h, cell lysates and immunoprecipitates were analyzed using SDS-PAGE and immunoblotting with corresponding antibodies. **D** Immunofluorescent staining of endogenous GABARAP, GABARAPL1, LC3B, and exogenous G2E3 in G2E3 KO AsPC-1cells. G2E3KO AsPC-1 cells were transfected with plasmids encoding G2E3-FLAG. After 48 h, cells were fixed. G2E3-FLAG was stained with anti-FLAG antibody and the co-localization of the endogenous GABARAP, LC3B and GABARAPL1 were visualized under confocal microscope. Scale bar: 10 μm.

Next, we investigated the mechanism by which G2E3 regulates autophagic flux. We observed that G2E3 KO led to 1–2-fold increases in GABARAP, GABARAPL1 and LC3B levels (Fig. 3B). These phenotypes were partially rescued by transfection with C-terminally FLAG-tagged G2E3 plasmid (G2E3-FLAG) in AsPC-1 cells (Fig. 3A). In addition, in vivo co-immunoprecipitation (co-IP) assays showed that transiently expressed G2E3-FLAG associated with endogenous GABARAP and GABARAPL1, but not with ubiquitins (Supplementary Fig. 2C) or LC3B in HEK293T cells (Fig. 3C, left). This interaction was further validated in AsPC-1 cells, where G2E3-FLAG co-immunoprecipitated with endogenous GABARAP and GABARAPL1 (Fig. 3C, right). Confocal microscopy further revealed that in AsPC-1 G2E3 KO cells, G2E3-FLAG displayed more prominent colocalization with GABARAP and GABARAPL1 than with LC3B (Fig. 3D). Taken together, these findings indicate that G2E3 specifically interacts with GABARAP and GABARAPL1, but not with LC3B.

### G2E3 is required for autophagosome maturation

As G2E3 protein is involved in autophagy process, and it works in the late stage, thus we wondered whether G2E3 might regulate autophagosome maturation.

**Fig. 4:**
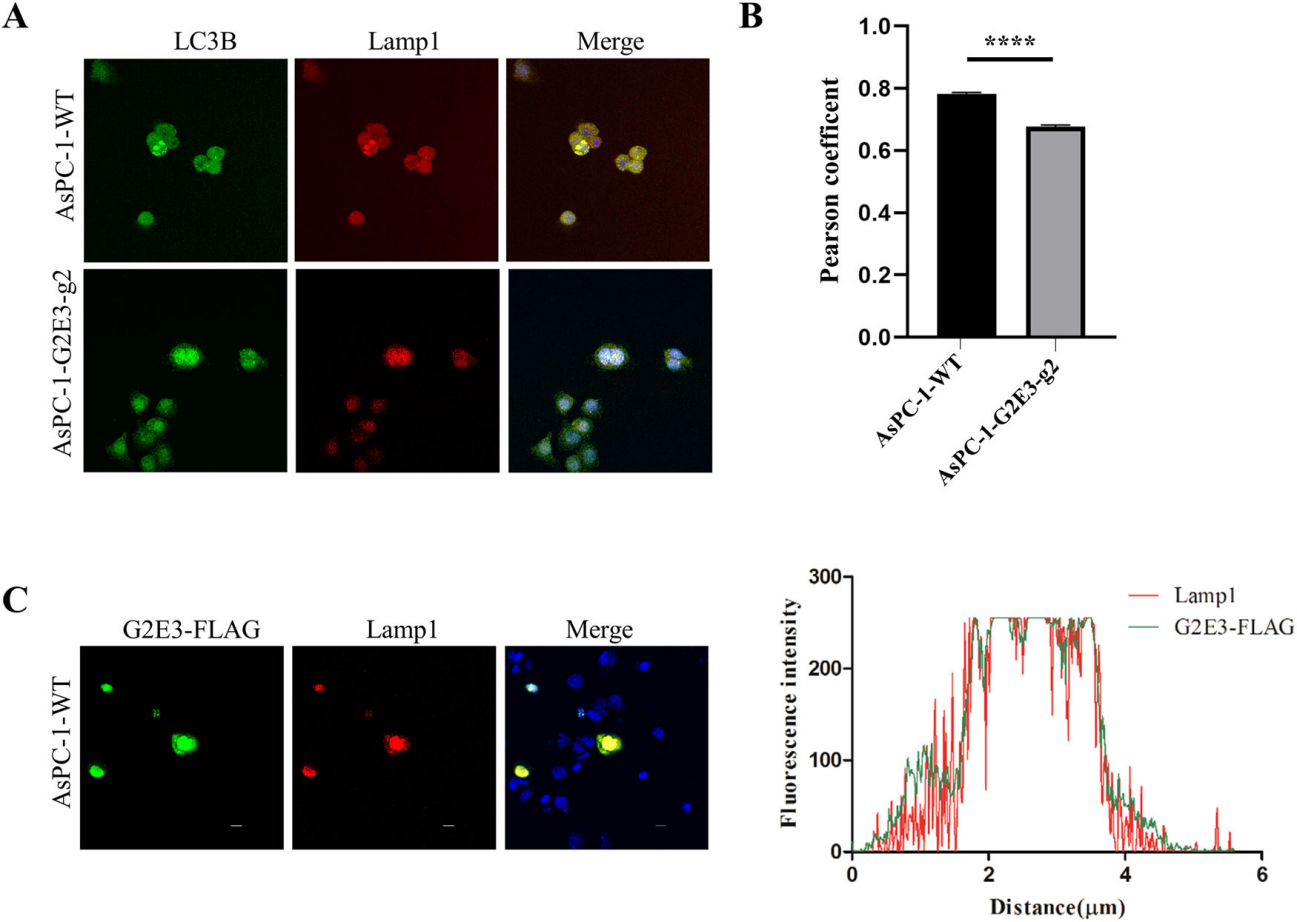
G2E3 regulates the fusion of autophagosome-lysosome. **A** WT or G2E3-KO AsPC-1 cells were fixed and stained with Lamp1 (red), LC3 (green), and DAPI (blue). Scale bar 10 μm. **B** The Pearson’s coefficients of LC3B overlap with Lamp1 in (**A**) were quantified using Fiji software. Mean ± SEM, *n* = 10. ****p < 0.0001. Significance was calculated with Student’s *t* test. **C** Co-localization analysis of endogenous Lamp1 and exogenously expressed G2E3-FLAG. Line plots exemplify degree of co-localization of G2E3 and Lamp1 signals.

As shown in Fig. 4A, B, the endogenous autophagosome marker LC3B co-localized with lysosome-associated member protein 1 (LAMP1) in WT AsPC-1 cells. However, the co-localization of LC3B with LAMP1 was attenuated following G2E3 KO, confirming that G2E3 plays a role in regulating autophagosome maturation. This result was supported by the confocal microscopy results that in Fig. 2F. Compared with WT AsPC-1 cells, G2E3 KO AsPC-1 cells showed a significant increase in the number of double-positive puncta (Fig. 2G) but a decrease in number of mCherry-only puncta (Fig. 2H). This observation indicates a large number of autophagosomes failed to fuse with lysosomes.

To further investigate the co-localization of G2E3 with Lysosomes, we transiently transfected AsPC-1 cells with G2E3-FLAG plasmids and found that G2E3 co-localized with Lamp1. Overall, these results demonstrated that G2E3 is responsible for autophagosome maturation.

### G2E3 KO suppresses the invasion and migration of pancreatic cancer cells in vitro

Given that G2E3 is involved in autophagy regulation, and dysfunctional autophagy is critical for tumor progression, and previous studies have implicated G2E3 in breast cancer. We conducted functional experiments to investigate the carcinogenic role of G2E3 in pancreatic cancer cells.

**Fig. 5:**
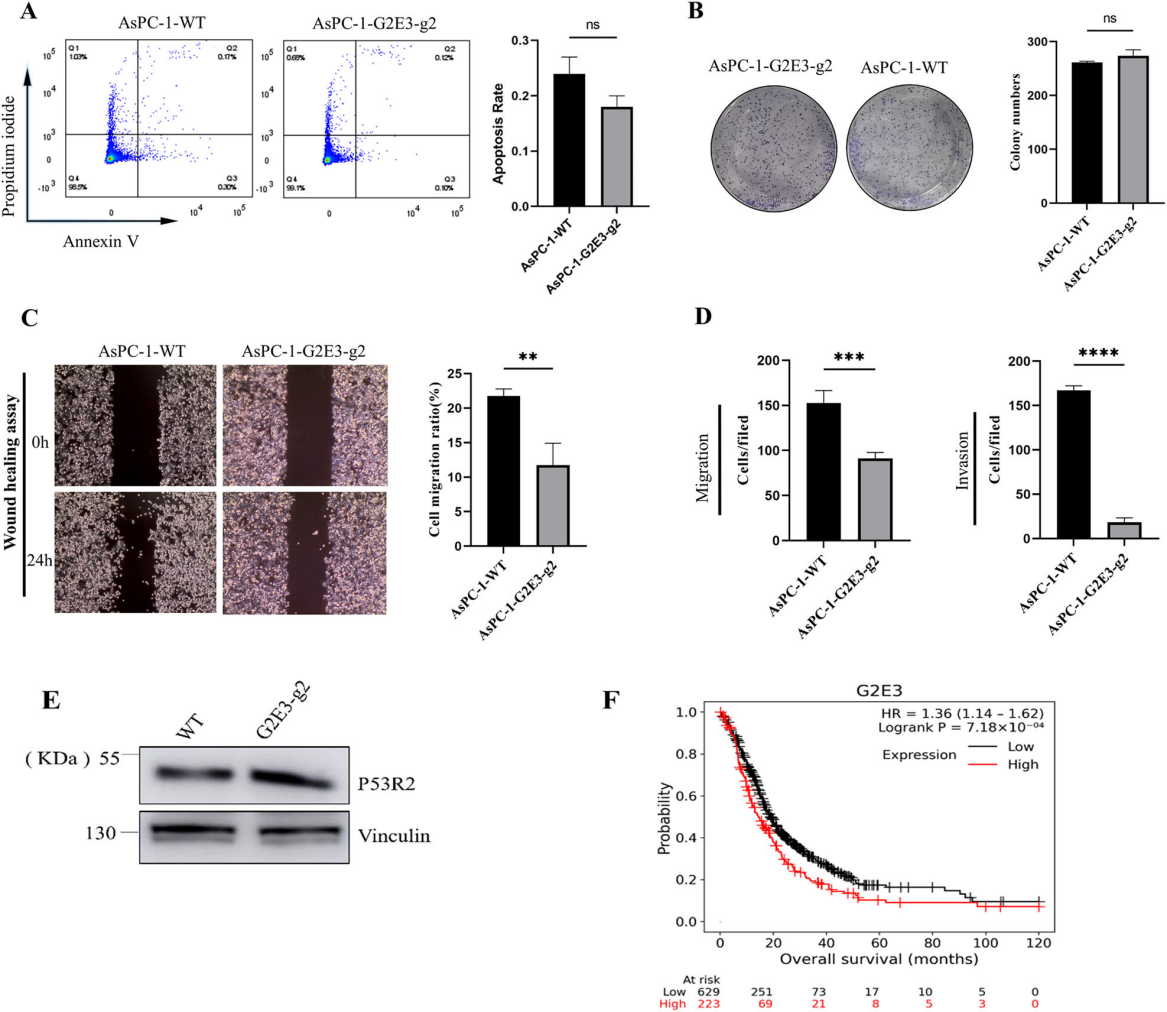
G2E3 KO inhibits pancreatic cancer cells migration and invasion. **A** Flow-cytometry-based apoptosis analysis of WT and G2E3 KO AsPC-1 cells. Data were presented as means of three independent experiments. n.s. not significant by Student’s *t* test. **B** Representative images from the colony formation assay (left picture) and colony number analysis (right picture). All experiments were performed in triplicate and data were presented as the mean ± SEM. **C** Wound healing assay to explore the migration effect of WT and G2E3 KO AsPC-1 cells, and calculate its migration rate. **p < 0.01. **D** Parental WT and G2E3 KO AsPC-1 cells were subjected to transwell migration and invasion assays. Error bars, Mean ± SEM, *n* = 10. ***p < 0.001, ****p < 0.0001. **E** Western blot analysis of p53R2 in G2E3 KO or parental WT AsPC-1 cells. Compared to the control, p53R2 was significantly enhanced. **F** The correlation between G2E3 expression and prognosis of patients with PDAC (HR = 1.36) by Kaplan–Meier survival analysis. HR hazard rate. Low expression of G2E3 is associated with longer survival of PDAC patients.

First of all, flow cytometry analysis results showed no significant difference in apoptosis between WT and G2E3 KO AsPC-1 cells (Fig. 5A). Similarly, colony formation assays revealed no difference in clonogenic potential between G2E3 KO and WT cells (Fig. 5B). By contrast, wound healing, migration and invasion assays demonstrated striking effects: G2E3 KO AsPC-1 cells exhibited a 50% reduction in migration capacity (wound healing assay, *p* < 0.01, Fig. 5C), a 41% reduction in transwell migration capability (*p* < 0.001, Fig. 5D left) and an 80% decrease in invasion (*p* < 0.0001, Fig. 5D right).

To validate these findings in vivo, we analysed gene array data from 852 patients with Kaplan–Meier survival plots (https://kmplot.com/analysis/index.php?p=home) [10]. As depicted in Fig. 5F, patients with low expression of G2E3 had significantly longer survival than patients with high G2E3 expression.

The EMT (epithelial-mesenchymal transition) process has been widely implicated in invasion, metastasis, tumor heterogeneity, and therapy resistance across multiple tumor types [11]. To explore whether G2E3 regulates EMT process in PDAC, we used western blot analysis to assess the expression of N-cadherin, E-cadherin and Vimentin in PDAC cell line AsPC-1. The results showed no significant differences between G2E3 KO and WT AsPC-1 cells (Supplementary Fig. 2D). Given that KRAS and TP53 mutations are major drivers of PDAC [12], and p53R2 overexpression has been shown to reduce cancer cells invasion and migration in both p53 WT and mutant cancer cell line [13], we evaluated the expression of RAS-G12D and p53R2 in WT and G2E3 KO AsPC-1 cells. Notably, the expression of RAS-G12D remained unchanged between groups, whereas the expression of p53R2 was upregulated in G2E3 KO AsPC-1 cells compared to WT cells.

Collectively, these findings suggest that G2E3 KO inhibits pancreatic cancer progression, potentially by modulating p53R2 expression.

## Discussion

Recently, CRISPR-based library screens have demonstrated significant advantages in identifying novel autophagy regulators in mammalian cells [7, 14]. Here, we integrated CRISPR screening with FACS sorting to identify ubiquitination-related proteins involved in autophagy regulation. Prior to library transduction, two rounds of FACS sorting were conducted to select Torin1-sensitive reporter cells. Notably, Torin1 demonstrates a more potent capacity for autophagy induction compared to starvation or rapamycin, a characteristic that significantly enhances the accuracy of library screening. In the initial screening, we isolated the top 3% of GFP-positive cells and maintained this gating criterion across an additional four rounds of sorting. This approach successfully identified the positive control ATG7 and other known autophagy components as primary hits, providing robust validation of our screening methods effectiveness. In follow-up studies, we identified G2/M-Phase specific E3 ubiquitin protein ligase [15] (G2E3) as a novel positive autophagy regulator in multiple human cell line, these findings establish a new paradigm for the interaction between E3 ligases and autophagic pathway.

G2E3 is predominantly expressed in the central nervous system and during the early stage of limb buds formation in developing embryos [15], playing a vital role in averting apoptosis in the initial stage of embryonic development. Additionally, our studies indicate that G2E3 KO leads to the inhibition of autophagic flux. Mechanistically, G2E3 interacts specifically with GABARAP and GABARAPL1 rather than LC3B. GABARAPs bind to PLEKHM1 via the LIR motif, and PLEKHM1 has been shown to drive autophagosome–lysosome fusion by recruiting the HOPS complex during autophagy, thereby positively regulating fusion of autophagosome and lysosome [6]. Beyond this pathway, studies have shown that GABARAPs recruit the phosphatidylinositol-4-kinase PI4KIIα (a lipid kinase) to autophagosomes, where it generates phosphatidylinositol 4-phosphate (PI4P). The production of PI4P on autophagosomes is critical for their fusion with lysosomes [16, 17]. However, the mechanisms underlying GABARAP-PI4KIIα-PI4P-dependent autophagosome- lysosome fusion remain to be investigated [17].

Furthermore, the attenuated colocalization of LC3B with LAMP1 in G2E3 KO cell lines further corroborates this conclusion. Notably, in G2E3 KO cells (e.g., AsPC-1 cells and A549 cells), LC3B-II levels are significantly increased, likely due to failed autophagosome-lysosome fusion leading to LC3B-II accumulation. The detailed mechanisms by which G2E3 regulates core components of the fusion machinery, such as PLEKHM1, RAB7, and syntaxin17, remain to be elucidated.

ATG8 family members exhibit highly similar structures but differ in their protein interaction profiles [16]. For instance, the adaptor protein PLEKHM1 contains a GABARA-interacting motif (GIM, [W/F]-[V/I]-X2-V), which binds more tightly to GABARAP than LC3s [18]. Although G2E3 functions analogously to PLEKHM1, a key distinction lies in their interaction specificity: while PLEKHM1 engages all ATG8 family members [6], G2E3 exclusively interacts with GABARAPs and co-localizes with lysosomes.

RING-like ubiquitin ligases play a critical role in autophagy regulation by controlling protein ubiquitination for autophagic degradation [19]. Recent evidence indicates that RING E3 ligases, including TRIMs [20] and RNFs [21], mediate ubiquitination of core autophagic proteins to modulate autophagic activity. G2E3 has been identified as a dual-function ubiquitin ligase containing a catalytically inactive HECT (homologous to E6-associated protein) domain [15] and two distinct RING-like domains that catalyze K48-linked polyubiquitination. Future studies are needed to clarify whether G2E3 regulates autophagy via its RING-like ubiquitin ligase domains.

Our colony formation assay and flow cytometry analysis demonstrated that G2E3 KO exerted no significant effect on the proliferation rate or apoptosis of AsPC-1 cells in vitro. This finding contradicts with previous studies, which reported that knockdown of G2E3 may promote apoptosis and suppress cancer cell proliferation [22].

Researchers have demonstrated that G2E3 expression is higher in breast cancer tissues than in normal breast tissues, and high G2E3-positive expression is associated with poorer survival outcomes in breast cancer patients [22]. However, the expression and function of G2E3 in pancreatic cancer have yet to be demonstrated. In biologic studies, we found that G2E3 KO markedly decreased pancreatic cancer cells migration and invasion in vitro, additionally, the Kaplan-Meier plotter analysis suggested that low expression of G2E3 was associated with longer survival of pancreatic cancer patients.These findings indicate that G2E3 may function as an oncogene in pancreatic cancer.

The role of p53R2 in cancer progression is still debated. Previous studies have shown that p53R2 (p53-inducible protein and homolog of the ribonucleotide reductase R2 subunit) expression is negatively correlated with cancer cell invasion. In both p53 WT and mutant cell lines, upregulated p53R2 expression suppressed invasion potential, whereas decreased p53R2 promoted it [13]. In the present, our data showed that G2E3 KO leading to p53R2 overexpression in pancreatic cancer cells. Thus, these results indicate that G2E3 KO suppresses pancreatic cancer progression, potentially by modulating p53R2 expression.

While dysregulated autophagy has been shown to promote pancreatic cancer progression by influencing tumor cell growth, therapeutic resistance and metastasis [1, 23], the precise mechanism by which G2E3 influences pancreatic cancer progression remains undefined-whether through its oncogenic activity or autophagy-dependent pathways. It is also plausible that G2E3 exerts its effect on pancreatic cancer progression via both its oncogenic role and its regulation of autophagic processes. Future studies are warranted to elucidate these underlying mechanisms. The absence of G2E3-specific inhibitors precluded us from investigating the in vivo therapeutic potential of targeting G2E3 in pancreatic cancer models.

Collectively, our findings showed that G2E3 is an autophagy regulator that facilitates autophagosome-lysosome fusion by scaffolding GABARAPs and we also identified a new molecular mechanism underlying pancreatic cancer progression, suggesting that, G2E3 could be targeted for pharmacologic treatment of pancreatic cancer and other diseases associated with dysfunctional autophagy.

## Materials and methods

### Antibodies

Antibody information: For western blot and co-IP, rabbit anti-FLAG (Cell Signaling Technology Cat# 14793, RRID:AB_2572291), mouse anti-FLAG (Cell Signaling Technology Cat# 8146, RRID:AB_10950495), anti-GABARAP (Cell Signaling Technology Cat# 13733, RRID:AB_2798306), anti-GABARAPL1 (Cell Signaling Technology Cat# 26632, RRID:AB_2798928), anti-GAPDH (Cell Signaling Technology Cat# 2118, RRID:AB_561053), anti-LC3B (Cell Signaling Technology Cat# 2775, RRID:AB_915950), anti-ubiquitin (Cell Signaling Technology Cat# 3936, RRID:AB_331292), anti-Vinculin (Cell Signaling Technology Cat# 13901, RRID:AB_2728768), anti-rabbit IgG, HRP-linked (Cell Signaling Technology Cat# 7074, RRID:AB_2099233), anti-mouse IgG, HRP-linked (Cell Signaling Technology Cat# 7076, RRID:AB_330924), anti-E-Cadherin (Cell Signaling Technology Cat# 3195, RRID:AB_2291471), anti-Vimentin (Cell Signaling Technology Cat# 5741, RRID:AB_10695459), anti-N-Cadherin (Cell Signaling Technology Cat# 13116, RRID:AB_2687616), anti-RAS(Cell Signaling Technology Cat# 3965, RRID:AB_2180216), anti-RAS-G12D(Cell Signaling Technology Cat# 14429, RRID:AB_2728748) were bought from Cell Signaling Technology. Anti-SQSTM1 (Abcam Cat# ab91526, RRID: AB_2050336) was bought from Abcam, anti-p53R2(Novus Cat# IMG-5504) was bought from Novus.

For immunofluorescence, anti-LC3B (Cell Signaling Technology Cat# 3868, RRID:AB_2137707), anti-mouse IgG Alexa Fluor® 555 conjugate (Cell Signaling Technology Cat# 4409, RRID:AB_1904022), anti-rabbit Alexa Fluor® 555 conjugate (Cell Signaling Technology Cat# 19581, RRID:AB_2943221), anti-rabbit IgG Alexa Fluor® 488 conjugate (Cell Signaling Technology Cat# 4412, RRID:AB_1904025), anti-mouse IgG Alexa Fluor® 488 conjugate (Cell Signaling Technology Cat# 4523, RRID:AB_836889) were purchased from Cell Signaling Technology.

### Plasmids

pMD2.G was a gift from Didier Trono (Addgene plasmid # 12259; http://n2t.net/addgene:12259; RRID:Addgene_12259), psPAX2 was a gift from Didier Trono (Addgene plasmid # 12260; http://n2t.net/addgene:12260; RRID: Addgene_12260), FUW mCherry-GFP-LC3 was a gift from Anne Brunet (Addgene plasmid # 110060; http://n2t.net/addgene:110060; RRID:Addgene_110060), Ubiquitination-Related Proteins CRISPR Knockout Library (Addgene #174592; http://n2t.net/addgene:174592; RRID:Addgene_174592), pSpCas9(BB)-2A-Puro (PX459) V2.0 was a gift from Feng Zhang (Addgene plasmid # 62988; http://n2t.net/addgene:62988; RRID:Addgene_62988), G2E3-FLAG plasmids (Sino Biological Cat# HG22847-CF) and FLAG plasmids (Sino Biological Cat# CV012) were provided by Sino Biological.

### Cell culture

HEK293TN cells (BioCat cat. # LV900A-1-GVO-SBI, RRID: CVCL_UL49) were grown in DMEM medium containing 10% inactivated fetal bovine serum (FBS) (Gibco™ Cat# A3160501). A549 cells (RRID: CVCL_0023) were cultured with Dulbecco’s Modified Eagle’s Medium F-12 Nutrient Mixture (Gibco™ Cat# 2662266, 10% FBS). AsPC-1 cells (RRID: CVCL_0152) were grown in Roswell Park Memorial Institute Medium 1640 supplemented with 10% FBS. All the cells were routinely cultured at 37 °C with 5% CO_2_ incubator. And all cells were washed with DPBS (Gibco™ Cat# 14190094), detached with 0.25% trypsin-EDTA (Sigma Cat# T4049-100ML).

### Lentiviral generation and transduction

HEK293TN cells were used to generate lentivirus using Lipofectamine 3000 Transfection Reagent (Invitrogen Cat# 2743119).At 90% confluency, cells were co-transfected with 1.4 μg pMD2.G, 1.4 μg psPAX2 and 4.3 μg of the expression plasmid (either FUW mCherry-GFP-LC3 or the Ubiquitination-Related Proteins CRISPR Knockout Library). 6 h post-transfection, the medium was replaced with 12 mL of fresh medium containing10% FBS. 24 h after transfection, the supernatant was collected, centrifuged at 2000 rpm for 10 min, aliquoted and stored in −80 °C. For lentiviral transduction, 5 million WT AsPC-1 cells were seeded into the T75 culture flask.The following day, cells were infected with lentivirus expressing mCherry-GFP-LC3, followed by selection with 400 μg/mL zeocin screening(Gibco™ Cat#R25001)for 14 days. Cells were then detached using 0.25% trypsin-EDTA (Sigma Cat# T4049-100ML), washed twice with DPBS, and resuspended in FACS buffer. Double- fluorescent cells were sorted via FACS.The process of library transduction is described in detail in the library screening section.

### Confocal microscopy

AsPC-1cells were seeded onto slides (SPL Lifesciences, Cat# 30404) for 6 h prior to transfection. After 48 h, cells were washed three times with DPBS, fixed with 4% formaldehyde for 15 min, and permeabilized by 0.1% Triton X-100(Sigma-Aldrich, Cat# T9284) at room temperature for 30 min. Cells were then incubated with corresponding primary antibodies overnight at 4 °C. Following three washes with 1 × TBST, the samples were incubated for 1 h with the corresponding Alexa Fluor-conjugated secondary antibodies at room temperature. Nucleis were stained with DAPI (Invitrogen Cat# 62248).

Cells stably expressing mCherry-GFP-LC3 were seeded onto slides at low density. After 24 h, cells were treated with 100 nM Torin1 (MedChemExpress Cat# HY-13003) for 12 h, followed by 1 μM Chloroquine phosphate (MedChemExpress Cat# HY-17589) for 2 h. The cells were washed three times with DPBS, fixed with 4% formaldehyde, and permeabilized with 0.1% Triton X-100 at room temperature for 30 min. Following three washes with 1 × TBST, nuclei were stained with DAPI. Fluorescence was monitored by Leica confocal laser microscopy TCS SP8 (Manheim, Germany).

### Flow cytometry

Following library infection or Torin1 incubation, AsPC-1 cells were detached, pelleted and resuspended in FACS buffer. Fluorescence intensity changes were monitored using a BD Biosciences LSRII Flow Cytometer, and cells exhibiting increased GFP fluorescence were sorted via FACS Aria II. Sorted cells transduced with the library were collected and cultured for subsequent rounds of sorting. The software FlowJo v10.8 was used to analyze the flow cytometry data.

### Immunoprecipitation and immunoblotting

AsPC-1 cells and HEK293TN cells were seeded in 6-well plates (1 × 10^6^ cells/well), and transfected with 2.5 μg of G2E3-FLAG plasmids using the Lipofectamine 3000 transfection kit, with empty FLAG plasmids as a control. After 48 h, cells were washed with PBS and lysed on ice for 60 min in RIPA buffer (Thermo Fisher Scientific Cat# 26149) supplemented with protease and phosphatase inhibitors (Thermo Fisher Scientific Cat# 78442). Cytosolic supernatants were collected by centrifugation at 13,000 × *g* for 30 min at 4 °C, and protein concentrations were determined using the BCA Protein Assay Kit (Thermo Fisher Scientific Cat# 23250) according to the manufacturer’s instructions. Equal amounts of protein were loaded onto 4–12% NuPAGE Bis-Tris protein gel (Thermo Fisher Scientific Cat# NP0322BOX). Proteins were then transferred onto polyvinylidene fluoride (PVDF) membranes (AmershamTM Hybond Cat# GE10600022), which were blocked with 5% slim milk in Tris-buffered saline with Tween 20(TBST) for 1 h at room temperature. Immunoprecipitation was conducted using Pierce Co-Immunoprecipitation (Co-IP) Kit (Thermo Scientific Cat# 26149). Membranes were incubated with primary antibodies overnight at 4 °Covernight, followed by HRP-linked secondary antibodies. Signals were developed using Signal Fire TM ECL Reagent (Cell Signaling Technology Cat# 6883S) and imaged using the Amersham Imager 600 system (Pittsburgh, USA). The intensities of bands were analyzed using ImageJ (National Institutes of Health, USA).

### CRISPR-Cas9 KO screen with human ubiquitination library

In this study, we used a human ubiquitination-related proteins CRISPR KO library to induce genomic mutations in AsPC-1-mCherry-GFP-LC3 cell line. 5.47 million AsPC-1-mCherry-GFP-LC3 cells were seeded in a T175 culture flask 12 h prior to infection. Cells were incubated with lentiviral supernatants at varying concentrations (supplemented with 1 μg/mL polybrene) for 24 h, parallel control cells were incubated with complete medium (without viral supernatant) at an identical cell density. Normal medium was then replaced with fresh medium containing 5ug/mL puromycin and cells were cultured for another two days. Exchange the normal medium with fresh medium containing 5 μg/mL puromycin. Three days later, when all control cells (without virus) were killed, the multiplicity of infection (MOI) was calculated using the formula: MOI = Number of cells surviving in puromycin-selected flask/number of cells in control flask. Based on this calculation, a viral volume corresponding to MOI = 0.25 was selected for subsequent experiments. Following puromycin selection, cells were continuously cultured until reaching a population of 5.5 million (providing 500-fold coverage of the library sgRNA). The first round of screen involved using a FACS Aria II Flow Cytometer to collect the top 3% of defective cells with increased GFP signals. The sorted cells were propagated to 5.5 million and subjected to subsequent rounds of sorting.

### Isolation of genomic DNA and next-generation sequencing

The genomic DNA was extracted using Nucleo Spin Blood L kit (Macherey-Nagel Cat# 740954.20) according to the manufacturer’s protocal. Followed, PCR was performed to amplify the DNA fragments encoding sgRNA with the primer: ACACTCTTTCCCTACACgACgCTCTTCCgATCTNNNNNTCTTgTggAAAggACgAAACACC (P5) and GTgACTggAgTTCAgACgTgTgCTCTTCCgATCTCCAATTCCCACTCCTTTCAAgACCT (P7). To achieve over 500-fold coverage, 10 μg of DNA was used in the PCR process. Q5 Master Mix (Biolabs Cat# M0494S) was used to conduct the 100-μL reactions containing 1 μL of genomic DNA, 5 μL of a 10 μM P5 primer solution, 5 μL of a 10 μM P7 primer solution. PCR cycling parameters were set as follows: initial denaturation at 95 °C for 1 min, 28 cycles of denaturation at 95 °C for 30 s, annealing at 53 °C for 30 s, extension at 72 °C for 30 s, and final extension at 72 °C for 10 min and hold at 4 °C. PCR products were diluted and subjected for deep Sequencing. Raw sequencing data were processed and analyzed using the MAGeCK software.

### CRISPR-Cas9 G2E3 mutant cell line

G2E3 was knocked out in AsPC-1, A549, HEK293TN cell lines using the CRISPR-Cas9 gene editing system. The targeting sequence for G2E3-sg1 (CACCGTGATAACTTCGTTTACATCG and AAACCGATGTAAACGAAGTTATCAC) and G2E3-sg2 (CACCGAACCAAGCGTTCTTACAACA and AAACTGTTGTAAGAACGCTTGGTTC) were cloned into pSpCas9 (BB)-2A-Puro (PX459) V2.0 vector. The sgRNAs target G2E3 were designed using the online CRISPR online tool (https://chopchop.cbu.uib.no/).

AsPC-1 cells were transfected with the G2E3 knock out plasmid three times using Lipofectamine 3000 transfection reagent (Cat # L3000015, Invitrogen, USA), whereas A549 and HEK293TN cells were transfected once. All cell lines underwent puromycin selection for three days following transfection. Western blotting and quantitative real- time PCR were then performed to assess the knockout efficiency. Monoclonal cells were isolated using 96-well plates, and the knockout effects were validated by qPCR and DNA sequencing.

### Genome mutation confirmation

The genomic DNA was isolated from cell lines using NucleoSpin®Blood Lkit (Machery Nagel Cat# 740954.20) according to the manufacturer’s protocol. PCR amplification for sequencing was conducted using Q5^®^ Hot Start High-Fidelity 2× Master Mix. The amplified PCR products were cloned into pMiniT 2.0 using the NEB PCR Cloning Kit (New England Biolabs, Cat# E1203S), and sequencing was performed by Eurofins Genomics.

The PCR primers used in this study were designed as follow:

hu-seq-G2E3-sg1-forward: 5‘- ACCCTTACCACAGCTGTTACA -3‘

reverse: 5‘- TGGGCGGGAGGGTAATACAT -3‘

hu-seq-G2E3-sg2-forward: 5‘- TTGTTGGGACCATCGACCTG -3‘

reverse: 5’TGGGAAGAAAAGCCTACTAATGT -3‘.

### RNA extraction and quantitative PCR

Cells were lysed, and total RNA was extracted using Kit NucleoSpin RNA PLUS (Macherey-Nagel Cat#740984.250). Reverse transcription was performed with High-Capacity cDNA Reverse-Transcription Kit (Applied Biosystems Cat# 00364942). Quantitative PCR (qPCR) was performed in triplicates using SYBR Green Master Mix (Applied Biosystems Cat# 4367659). Fold-changes were calculated using the 2^-ΔΔ^Ct method, with beta-actin (human) expression levels serving as the internal reference. Reactions were run on a QuantStudio 1 System (Thermo Fisher Scientific). Primers used for qPCR were: CTGGTGACTCACAGAACCTTG(forward)/TGCCTCTCTGCCAAATTCCAC (reverse) for G2E3 and CACCATTGGCAATGAGCGGTTC (forward)/AGGTCTTTGCGGATGTCCACGT (reverse) for beta-actin. All the primers were synthesized by Eurofins Genomics (Ebersberg, Germany).

### Apoptosis assay

The apoptosis assay was conducted using the APC Annexin V Apoptosis Detection Kit I (Cat# 556547, BD Pharmingen, San Diego, CA, USA) according to the manufacturer’s protocol. Briefly, AsPC-1 Cells were harvested, rinsed with PBS 3 times, resuspended in binding buffer, and stained with annexin V-FITC and PI for 15 min at room temperature. The apoptosis data were acquired using a BD Biosciences LSRII flow cytometer. Lastly, and subsequent data were analyzed with FlowJo v10.8 Software (BD Life Sciences, USA).

### The cell migration/invasion assay

The wound healing and transwell chamber assays were performed to determine the migratory and invasive ability of G2E3 KO AsPC-1 cells. For the wound healing assay, 1 × 10^6^ cells were seeded into 6-well plates and cultured until confluent. The 100 μL pipette tip was used to create a uniform scratch wound in each well of the plate, followed by washing with PBS to remove detached cells. Wound images were captured at 0 h and 24 h using a EVOS microscope. The gap width was measured using ImageJ software, and the migration rate was calculated using the formula: Migration rate (%) = [(Initial Gap Width-Final Gap Width)/Initial Gap Width] × 100%.

For the Transwell chamber assay, 24-well plates (Cat# REF353504, Corning, Germany) with inserts containing an 8.0 µm pore-size polycarbonate membrane (Cat# REF351152, Corning, Germany) were used. The inserts were pre-coated with a thin layer of Matrigel (Cat# 354230, Corning^®^) for the invasion assay or left uncoated for the migration assay. The lower chambers were filled with 700 μL of 10% FBS medium, while 5 × 10^4^ AsPC-1 cells resuspended in a 200 µL of FBS-free medium were inoculated into the upper chambers. The plates were incubated for 48 h (invasion assay) or 21 h (migration assay) at 37 °C.

Cells that invaded the matrigel and migrated to the opposite side of the insert membrane, were stained with NucSpot Live 488 Nuclear Stain (Cat# 40081, Biotium, USA) for 10 min. Pictures were taken by the Evos FL Auto 2 imaging system (Invitrogen, AMAFD2000).

### Colony formation assay

AsPC-1 cells were seeded in 6-well plates (3000 cells/well) and incubated at 37 °C, 5% CO_2_. After 7 days, cell culture medium was removed and cells were fixed with 4% formaldehyde solution for 15 min, then the formaldehyde was discarded. The plates were stained with 0.1% crystal violet for 30 min and washed with 1 × DPBS 5 times prior to image capturing. The number of colonies was counted.

### Statistical analysis

Graph Pad PrismVersion 9.5.1 and Graph Pad PrismVersion 5 were used to create graphs. For the Kaplan–Meyer survival analysis, statistical significance was evaluated using the Log-rank test. Data were presented as the mean ± SEM. Error bars indicate SEM. Each experiment was repeated at least three times. Comparisons between two groups were performed using an unpaired Student’s *t* test. *p* > 0.05 indicates no statistical significance (i.e., n.s.), *p < 0.05, **p < 0.01, ***p < 0.001, ****p < 0.0001.

## Supplementary information

Original Western blot pictures associated to the manuscript file

Original Western blot pictures associated to the manuscript file

## Data Availability

The datasets generated and/or analysed during the current study are available from the corresponding author on reasonable request.

## References

[1] Connor AA, Gallinger S. Pancreatic cancer evolution and heterogeneity: integrating omics and clinical data. Nat Rev Cancer. 2022;22:131–42. 34789870 10.1038/s41568-021-00418-1

[2] Levine B, Kroemer G. Biological functions of autophagy genes: a disease perspective. Cell. 2019;176:11–42. 30633901 10.1016/j.cell.2018.09.048 PMC6347410

[3] Su H, Yang F, Fu R, Li X, French R, Mose E, et al. Cancer cells escape autophagy inhibition via NRF2-induced macropinocytosis. Cancer Cell. 2021;39:678–93.e11. 33740421 10.1016/j.ccell.2021.02.016 PMC8119368

[4] Padman BS, Nguyen TN, Lazarou M. Autophagosome formation and cargo sequestration in the absence of LC3/GABARAPs. Autophagy. 2017;13:772–4. 28165849 10.1080/15548627.2017.1281492 PMC5388231

[5] Nakatogawa H. Mechanisms governing autophagosome biogenesis. Nat. Rev. Mol. Cell Biol. 2020;21:439–58. 32372019 10.1038/s41580-020-0241-0

[6] Nguyen TN, Padman BS, Usher J, Oorschot V, Ramm G, Lazarou M. Atg8 family LC3/GABARAP proteins are crucial for autophagosome–lysosome fusion but not autophagosome formation during PINK1/Parkin mitophagy and starvation. J Cell Biol. 2016;215:857–74. 27864321 10.1083/jcb.201607039 PMC5166504

[7] Jia R, Bonifacino JS. Negative regulation of autophagy by UBA6-BIRC6–mediated ubiquitination of LC3. eLife. 2019;8:e50034. 31692446 10.7554/eLife.50034 PMC6863627

[8] Hertel A, Alves LM, Dutz H, Tascher G, Bonn F, Kaulich M, et al. USP32-regulated LAMTOR1 ubiquitination impacts mTORC1 activation and autophagy induction. Cell Rep. 2022;41:111653. 36476874 10.1016/j.celrep.2022.111653

[9] Ebner P, Poetsch I, Deszcz L, Hoffmann T, Zuber J, Ikeda F. The IAP family member BRUCE regulates autophagosome–lysosome fusion. Nat Commun. 2018;9:599. 10.1038/s41467-018-02823-x PMC5807552 29426817

[10] Posta M, Győrffy B. Analysis of a large cohort of pancreatic cancer transcriptomic profiles to reveal the strongest prognostic factors. Clin Transl Sci. 2023;16:1479–91. 37260110 10.1111/cts.13563 PMC10432876

[11] Wu L, Liu Y, Deng W, Wu T, Bu L, Chen L. OLR1 is a pan-cancer prognostic and immunotherapeutic predictor associated with EMT and cuproptosis in HNSCC. IJMS. 2023;24:12904. 37629087 10.3390/ijms241612904 PMC10454104

[12] Hashimoto S, Furukawa S, Hashimoto A, Tsutaho A, Fukao A, Sakamura Y, et al. ARF6 and AMAP1 are major targets of *KRAS* and *TP53* mutations to promote invasion, PD-L1 dynamics, and immune evasion of pancreatic cancer. Proc Natl Acad Sci USA. 2019;116:17450–9. 31399545 10.1073/pnas.1901765116 PMC6717289

[13] Liu X, Zhou B, Xue L, Shih J, Tye K, Lin W, et al. Metastasis-suppressing potential of ribonucleotide reductase small subunit p53R2 in human cancer cells. Clin Cancer Res. 2006;12:6337–44. 17085643 10.1158/1078-0432.CCR-06-0799

[14] Shoemaker CJ, Huang TQ, Weir NR, Polyakov NJ, Schultz SW, Denic V. CRISPR screening using an expanded toolkit of autophagy reporters identifies TMEM41B as a novel autophagy factor. PLoS Biol*.* 2019;17:e2007044. 10.1371/journal.pbio.2007044 PMC6459555 30933966

[15] Brooks WS, Helton ES, Banerjee S, Venable M, Johnson L, Schoeb TR. G2E3 is a dual function ubiquitin ligase required for early embryonic development. J Biol Chem. 2008;283:22304–15. 18511420 10.1074/jbc.M803238200 PMC2494922

[16] Johansen T. Selective autophagy: ATG8 family proteins, LIR motifs and cargo receptors. J Mol Biol. 2020;432:80-103. 10.1016/j.jmb.2019.07.016 31310766

[17] Albanesi J, Wang H, Sun HQ, Levine B, Yin H. GABARAP-mediated targeting of PI4K2A/PI4KIIα to autophagosomes regulates PtdIns4P-dependent autophagosome-lysosome fusion. Autophagy. 2015;11:2127–9. 26391226 10.1080/15548627.2015.1093718 PMC4824573

[18] Zhao J, Li Z, Li J. The crystal structure of the FAM134B–GABARAP complex provides mechanistic insights into the selective binding of FAM134 to the GABARAP subfamily. FEBS Open Biol. 2022;12:320–31. 10.1002/2211-5463.13340 PMC8727931 34854256

[19] Cui D, Xiong X, Zhao Y. Cullin-RING ligases in regulation of autophagy. Cell Div. 2016;11:8. 27293474 10.1186/s13008-016-0022-5 PMC4902950

[20] Heo H, Park H, Lee MS, Kim J, Kim J, Jung SY, et al. TRIM22 facilitates autophagosome-lysosome fusion by mediating the association of GABARAPs and PLEKHM1. Autophagy. 2024;20:1098–113. 38009729 10.1080/15548627.2023.2287925 PMC11135824

[21] Xu C, Feng K, Zhao X, Huang S, Cheng Y, Qian L, et al. Regulation of autophagy by E3 ubiquitin ligase RNF216 through BECN1 ubiquitination. Autophagy. 2014;10:2239–50. 25484083 10.4161/15548627.2014.981792 PMC4502788

[22] Shen Y, Xue J, Yu J, Jiang Y, Bu J, Zhu T, et al. Comprehensive analysis of the expression, prognostic significance, and regulation pathway of G2E3 in breast cancer. World J Surg Oncol. 2022;20:398. 36517818 10.1186/s12957-022-02871-0 PMC9753372

[23] Yamamoto K, Iwadate D, Kato H, Nakai Y, Tateishi K, Fujishiro M. Targeting autophagy as a therapeutic strategy against pancreatic cancer. J Gastroenterol. 2022;57:603–18. 35727403 10.1007/s00535-022-01889-1 PMC9392712

