# Supplementary material for: CRISPR-Cas9 screening reveals G2E3 as a novel ubiquitin-linked factor controlling autophagosome-lysosome fusion and cancer cell progression
Source: Cell Death Discov. 2025 Oct 9;11:455. doi: 10.1038/s41420-025-02717-0 (PMC12511632; doi:10.1038/s41420-025-02717-0)
Supplement: Supplementary file 1 — Original Western blot pictures associated to the manuscript file [file 41420_2025_2717_MOESM1_ESM.docx]

**Supplementary**


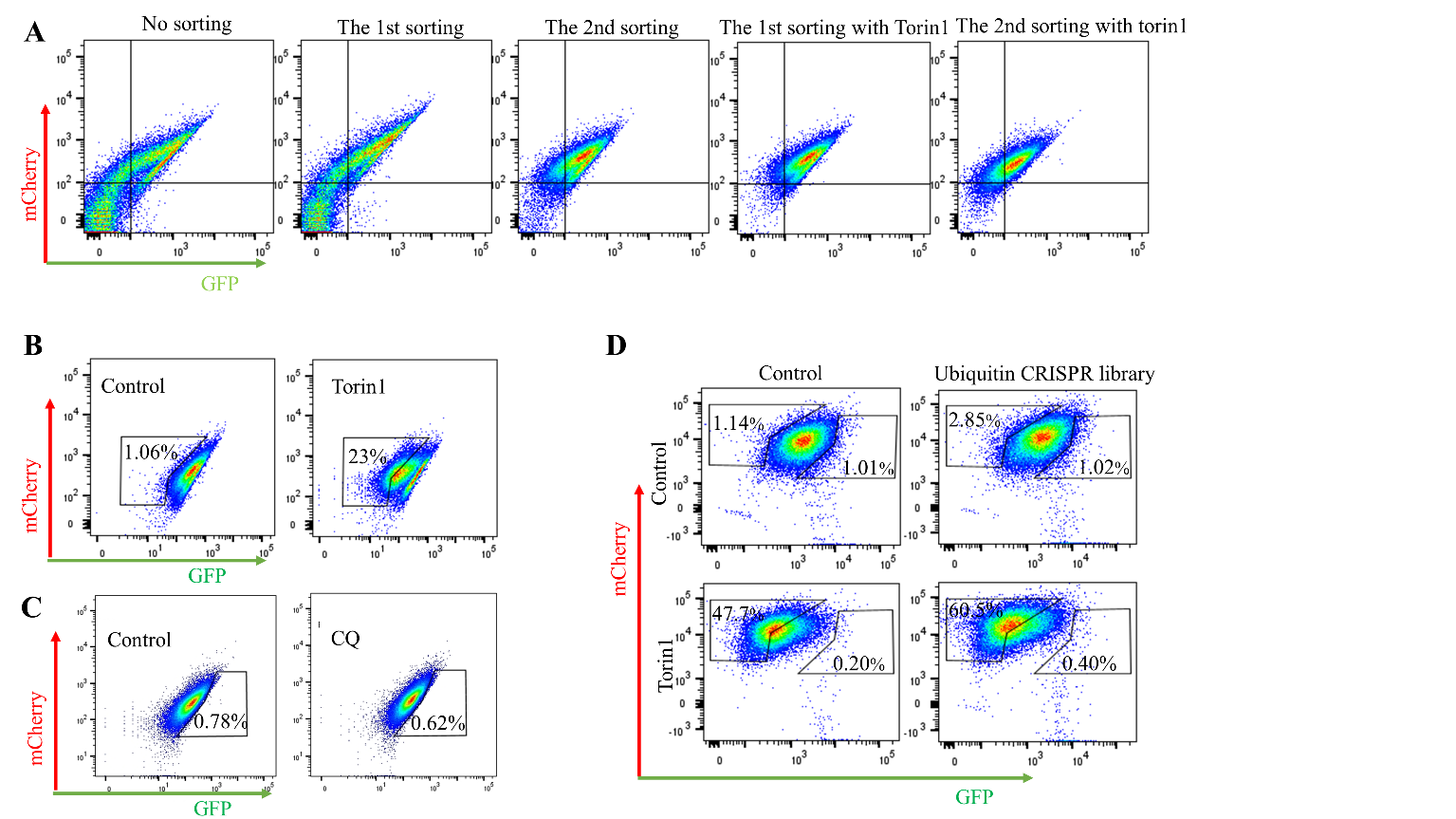


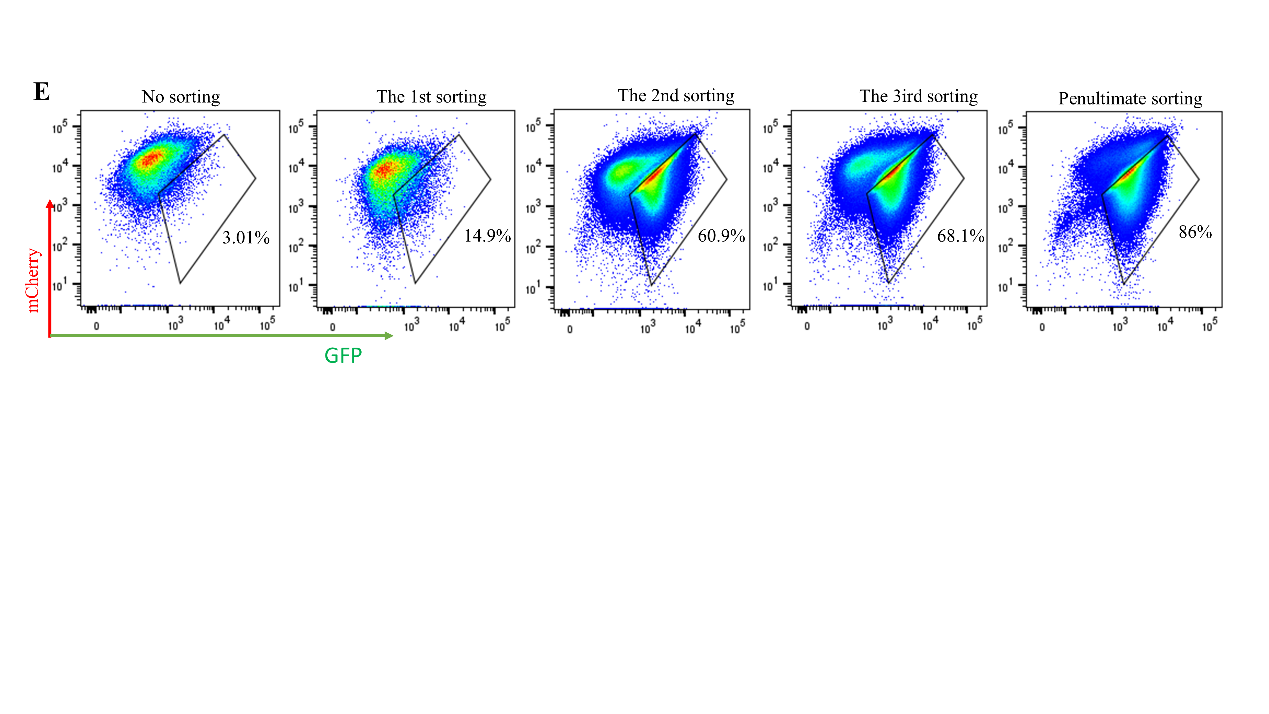


**Supplementry Figure 1. FACS sorting of AsPC-1 reporter cell line expressing mCherry-GFP-LC3.** (A) Torin1-sensitive reporter cells were screened. Following two rounds of FACS, the number of double-positive fluorescent cells increased. Subsequently, after 12 hours of Torin1 treatment, cells with low GFP signals were collected, expanded, and subjected to another round of FACS sorting. (B) AsPC-1 cells expressing mCherry-GFP-LC3 were incubated without (control) or with 100 nM Torin1 for 12 hours, and GFP/ mCherry fluorescence was measured by FACS. (C) AsPC-1 cells expressing mCherry-GFP-LC3 were incubated without (control) or with 2 μM CQ for 2 hours, and GFP/mCherry fluorescence was measured by FACS. (D) Gate setting for GFP-low or GFP-high cell populations in control and ubiquitin library transduced cells. Following autophagy induction, cells enriched in the GFP-high gate were selected, and the percentage of cells in the corresponding gates was indicated. (E) The population of defective cells in the gate increased as the FACs sorting progressed.


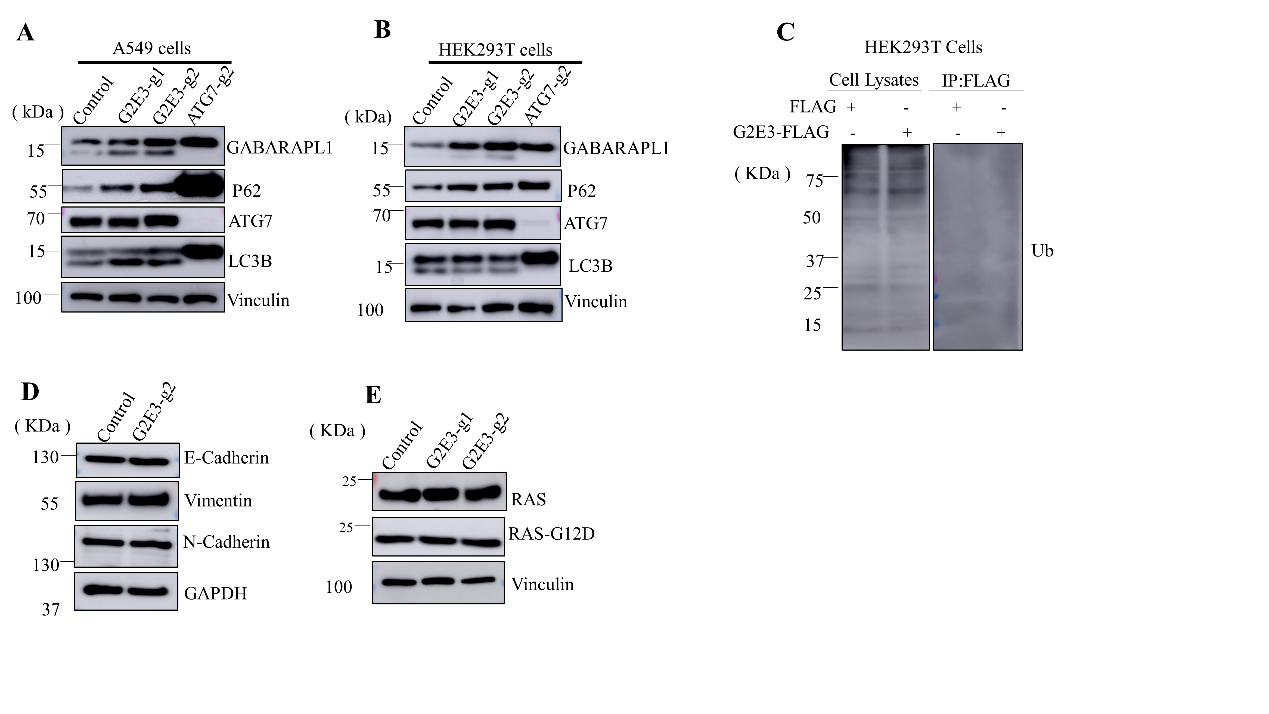


**Supplementary Figure 2**. (A) The expression of endogenous p62, LC3B and GABARAPL1 was analyzed in control, G2E3KO and ATG7 KO A549 cell line. (B)The expression of endogenous p62, GABARAPL1 and LC3B was analyzed in control, G2E3KO and ATG7 KO HEK293T cell line. (C) HEK293T WT cells were transfected with plasmids encoding G2E3-FLAG or plasmid FLAG. Cell lysates and immunoprecipitates were analyzed using SDS-PAGE and immunoblotting with ubiquitin antibody. (D) Western blot analysis of N-cadherin, E-cadherin, and vimentin expressions in G2E3 KO or control of AsPC-1 cells. (E) Western blot analysis of RAS and RAS-G12D in G2E3KO or control of AsPC-1 cells.
