# Supplementary material for: CRISPR-Cas9 screening reveals G2E3 as a novel ubiquitin-linked factor controlling autophagosome-lysosome fusion and cancer cell progression
Source: Cell Death Discov. 2025 Oct 9;11:455. doi: 10.1038/s41420-025-02717-0 (PMC12511632; doi:10.1038/s41420-025-02717-0)
Supplement: Supplementary file 2 — Original Western blot pictures associated to the manuscript file [file 41420_2025_2717_MOESM2_ESM.docx]

**Original Data**


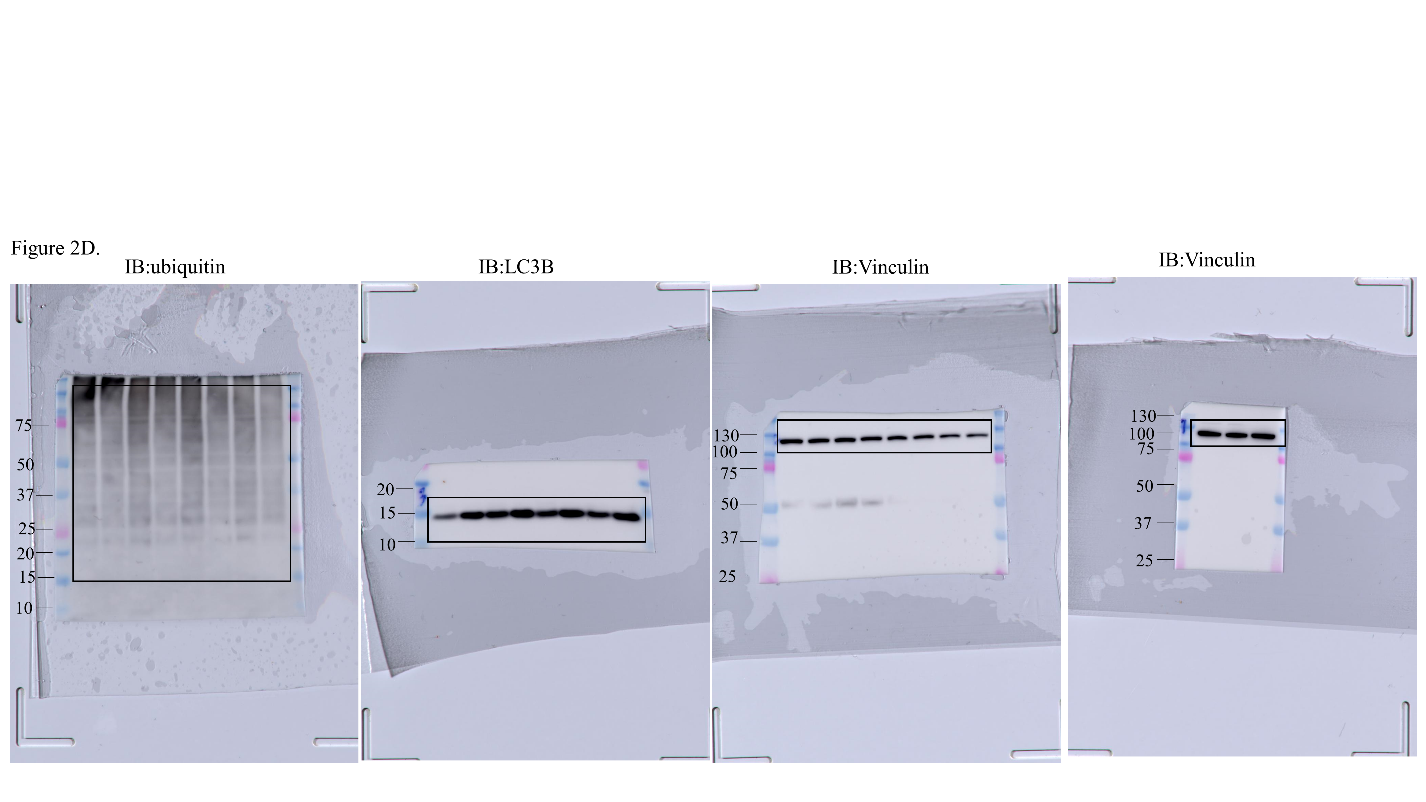


**Uncropped picture for Figure 2D.** The areas marked with boxes are presented in the indicated figures.


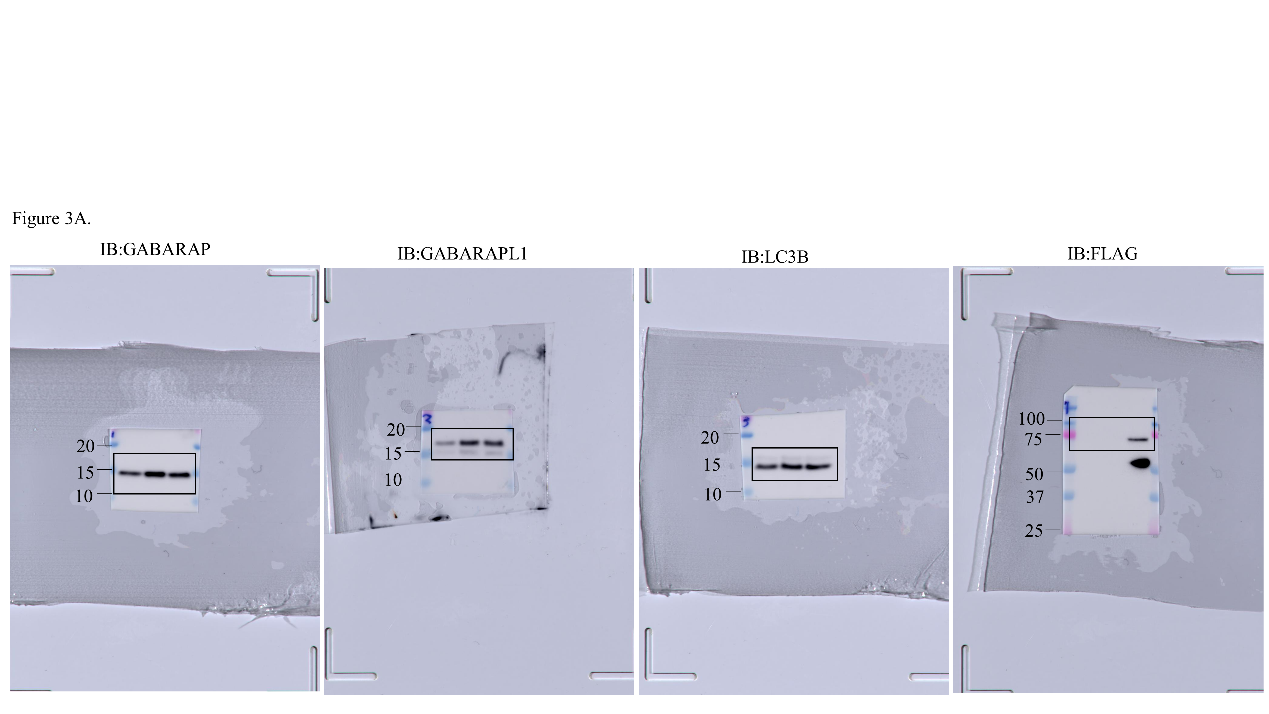


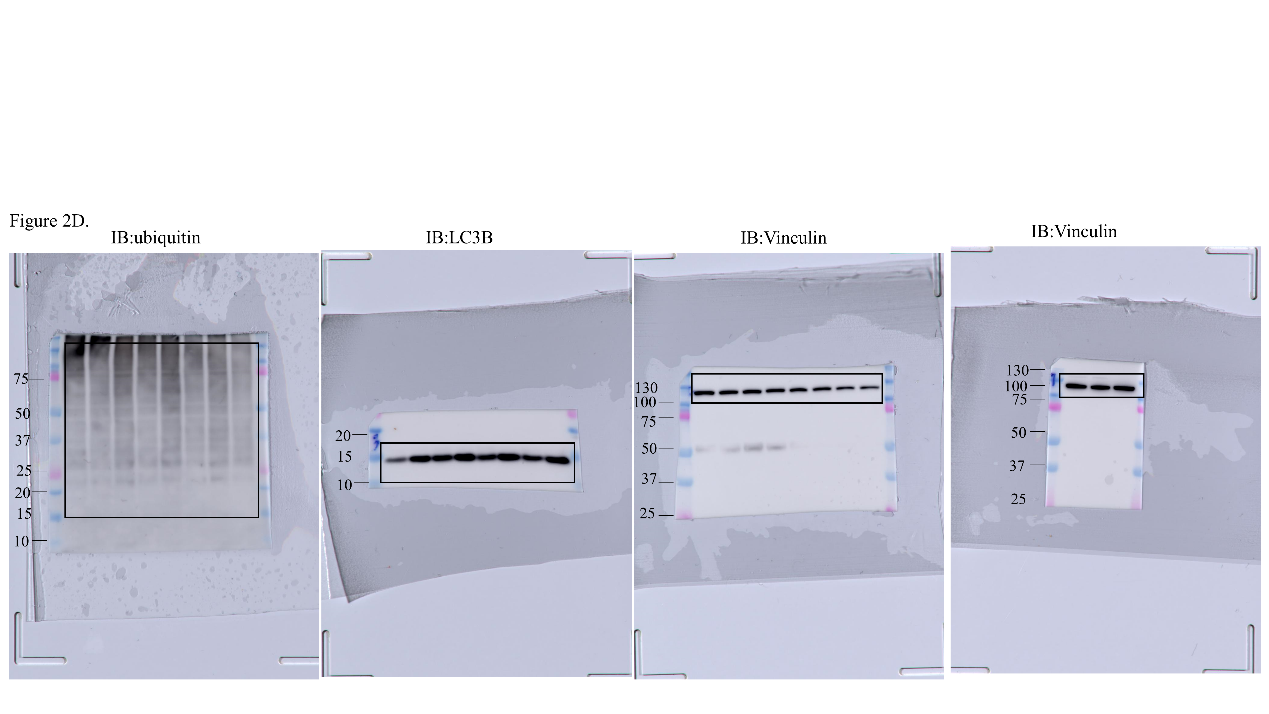


**Uncropped picture for Figure 3A**. The areas marked with boxes are presented in the indicated figures.


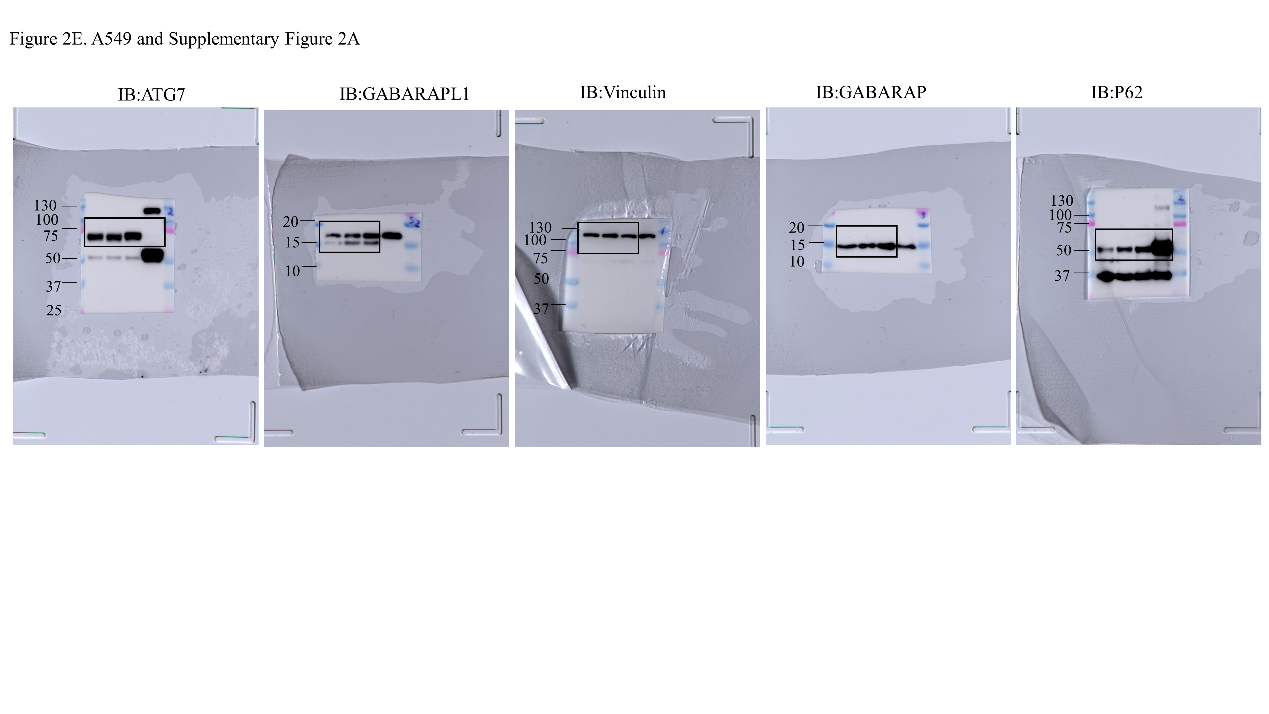


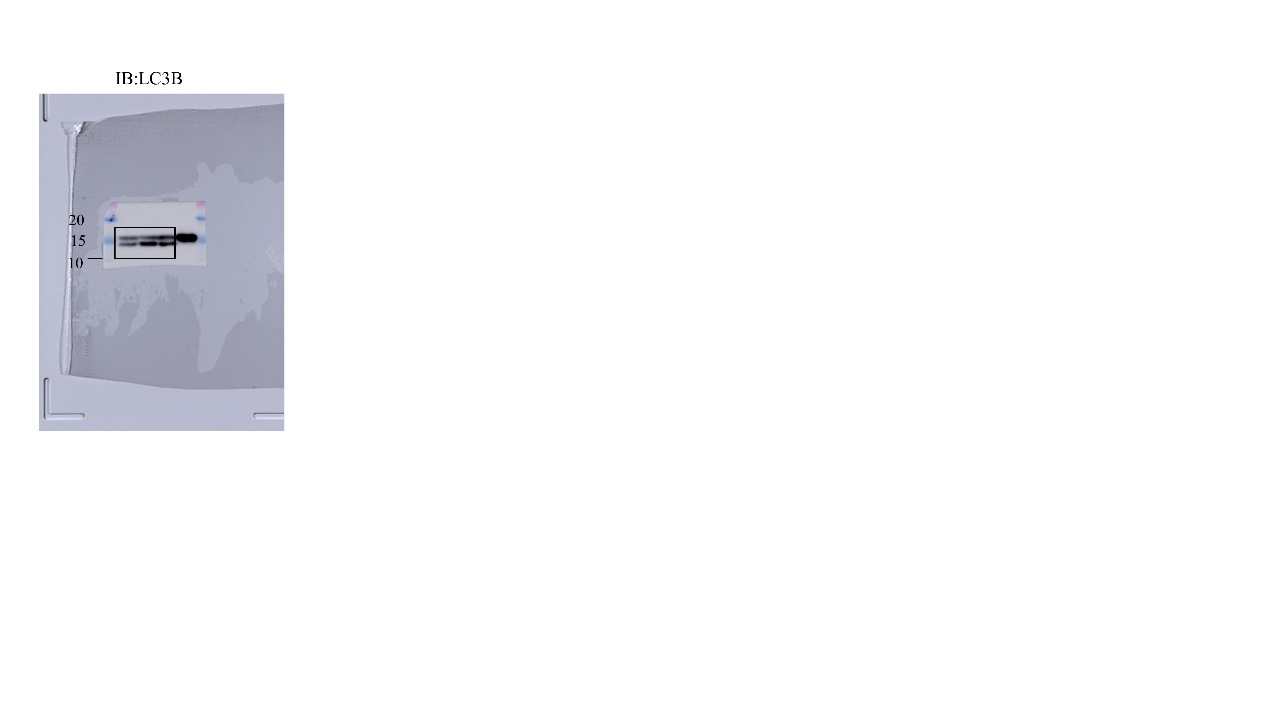


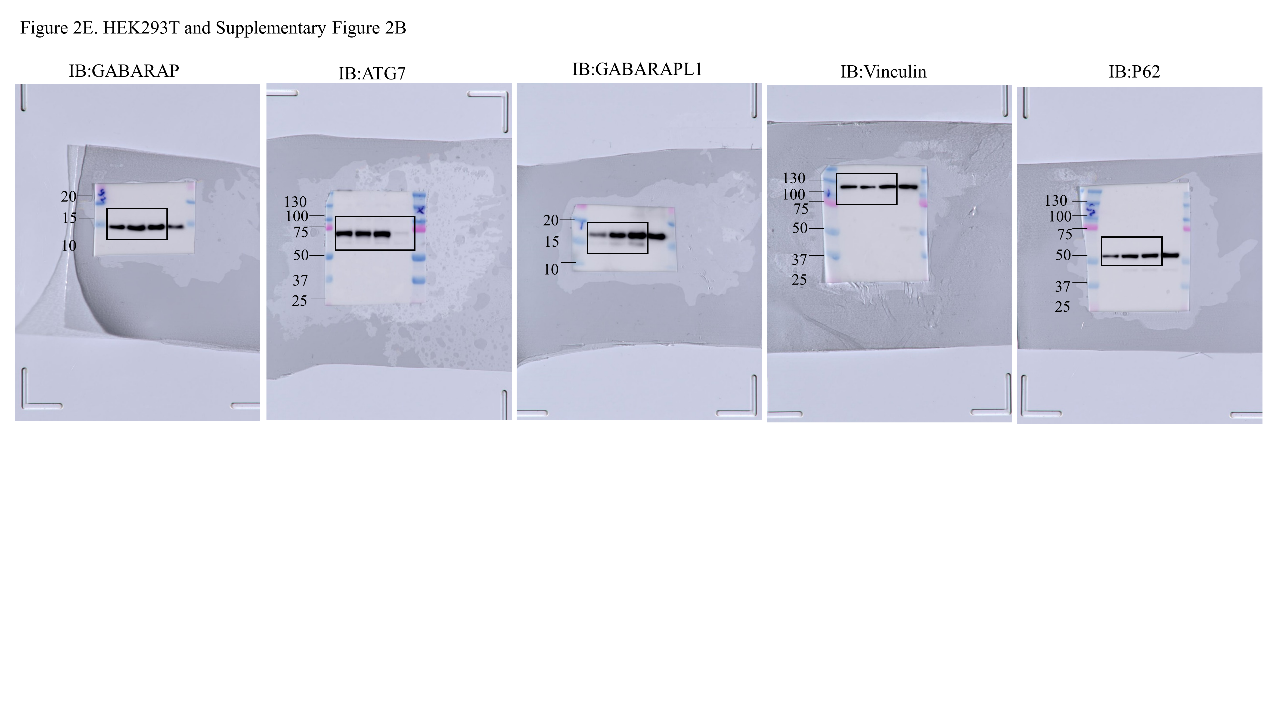

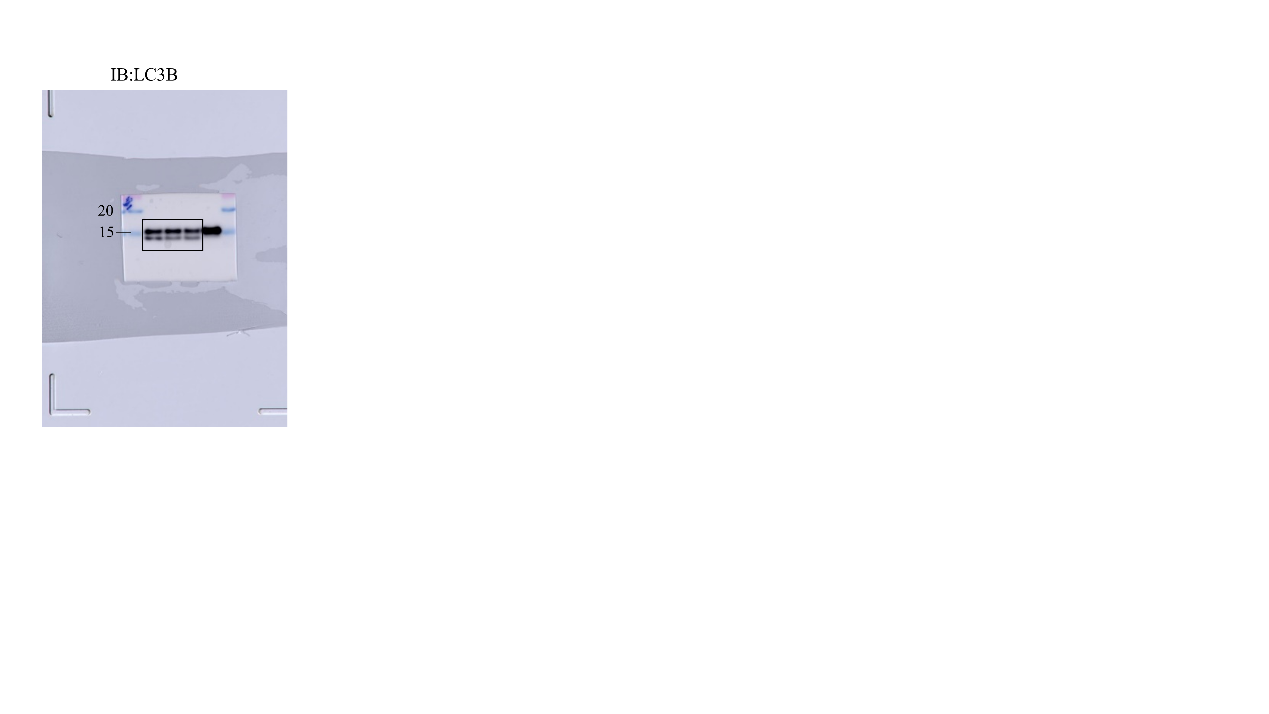


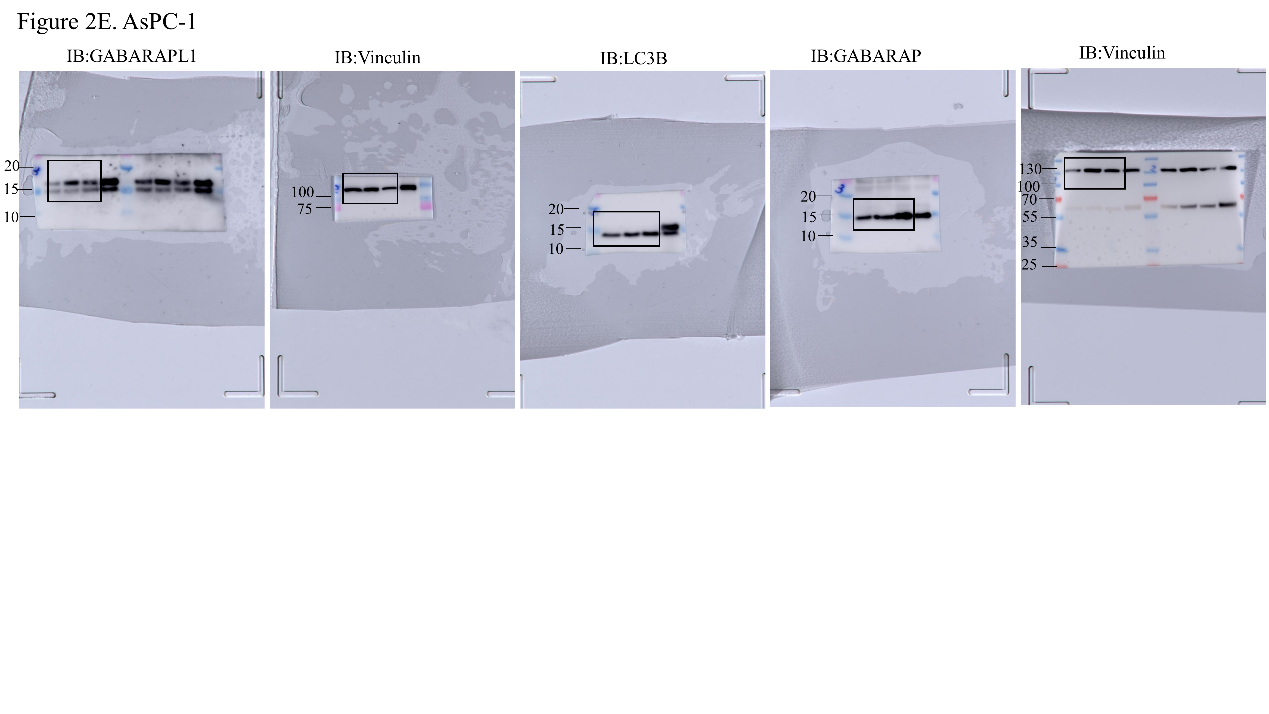


**Uncropped picture for Figure 2E and Supplementary Figure 2**. The areas marked with boxes are presented in the indicated figures.


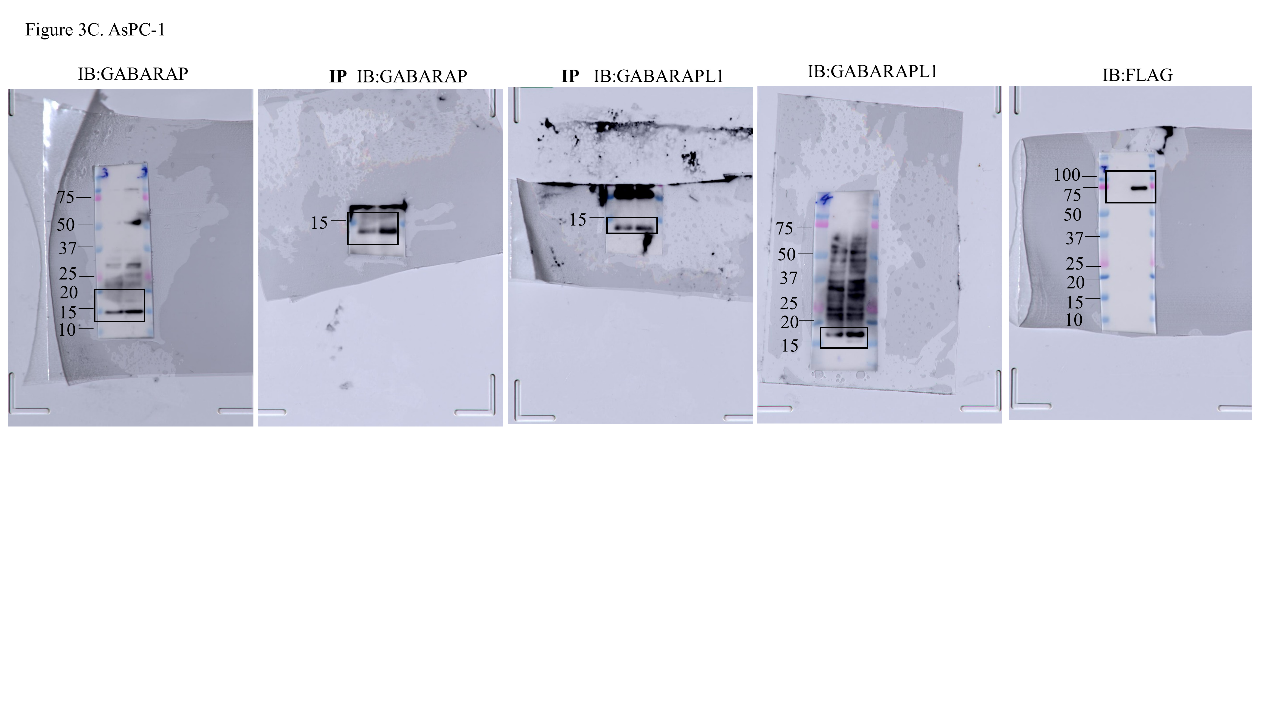


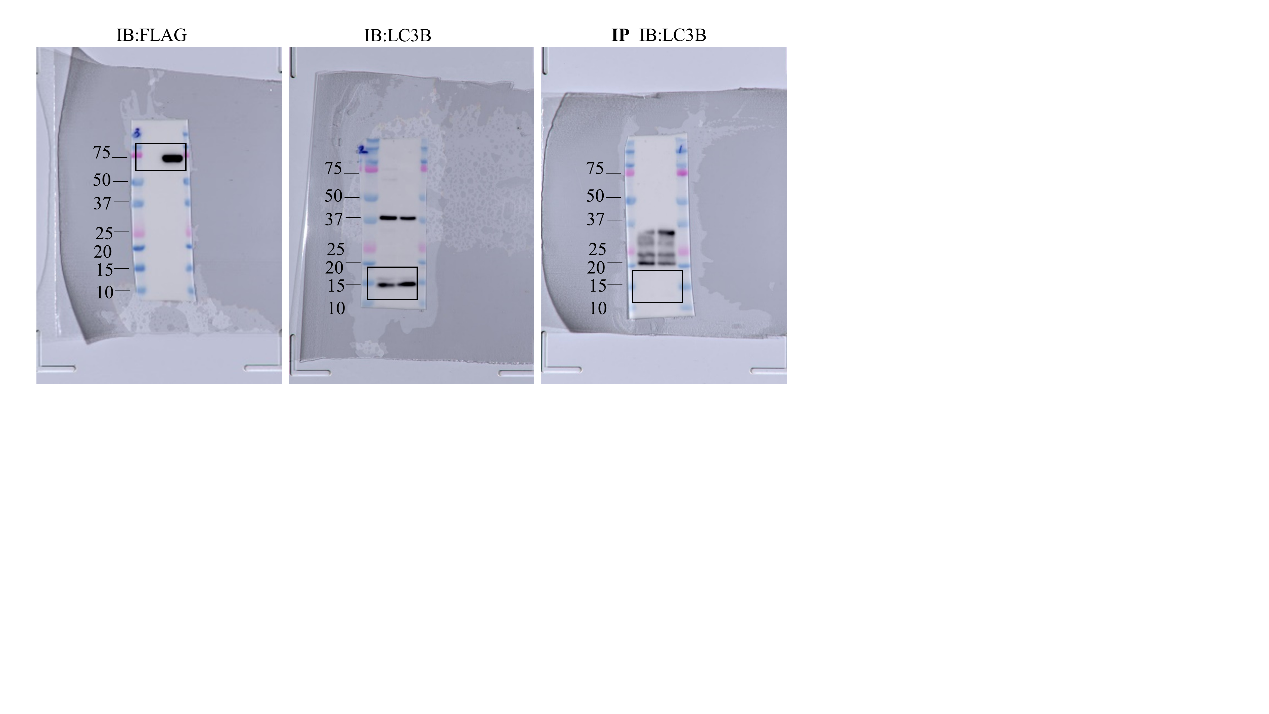


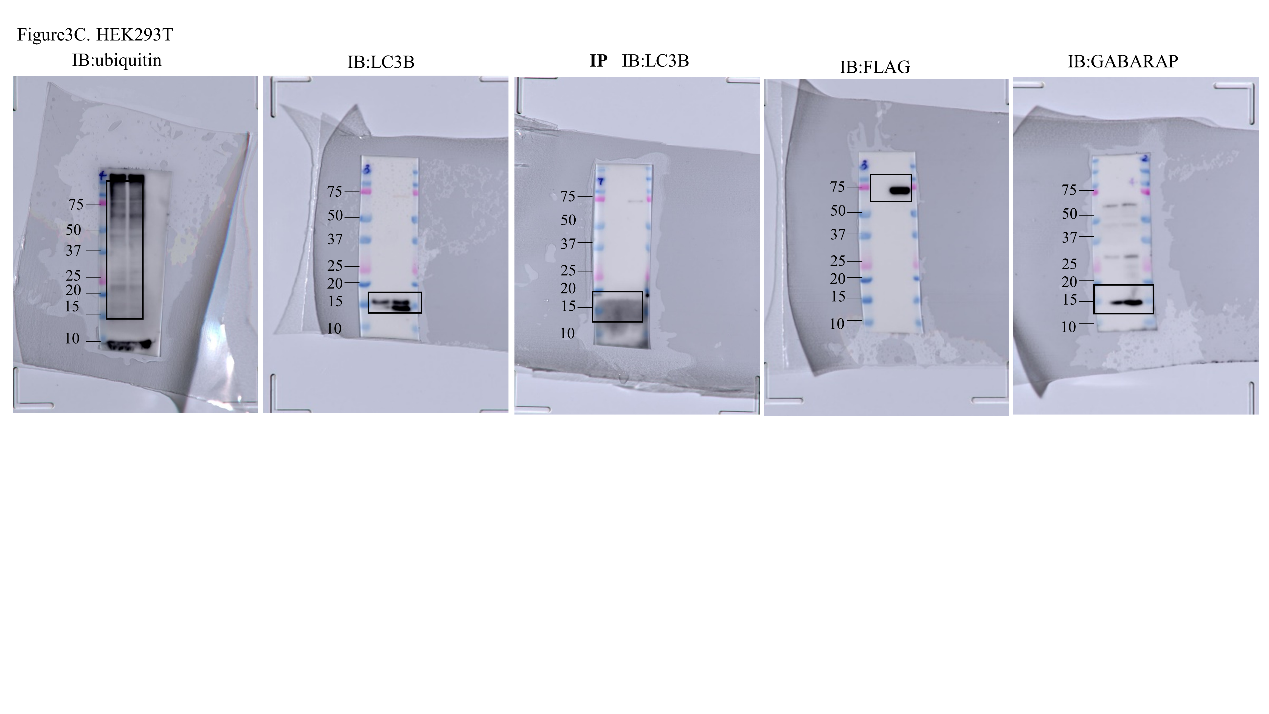


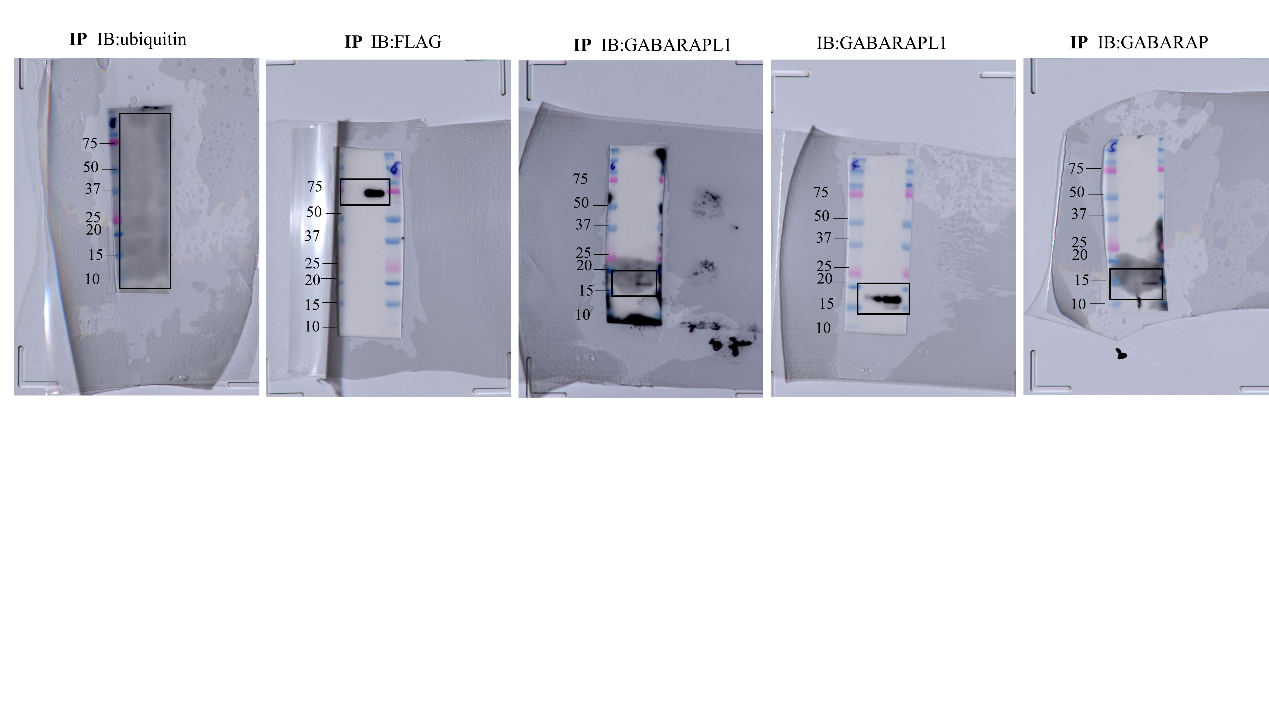


**Uncropped picture for Figure 3C**. The areas marked with boxes are presented in the indicated figures.


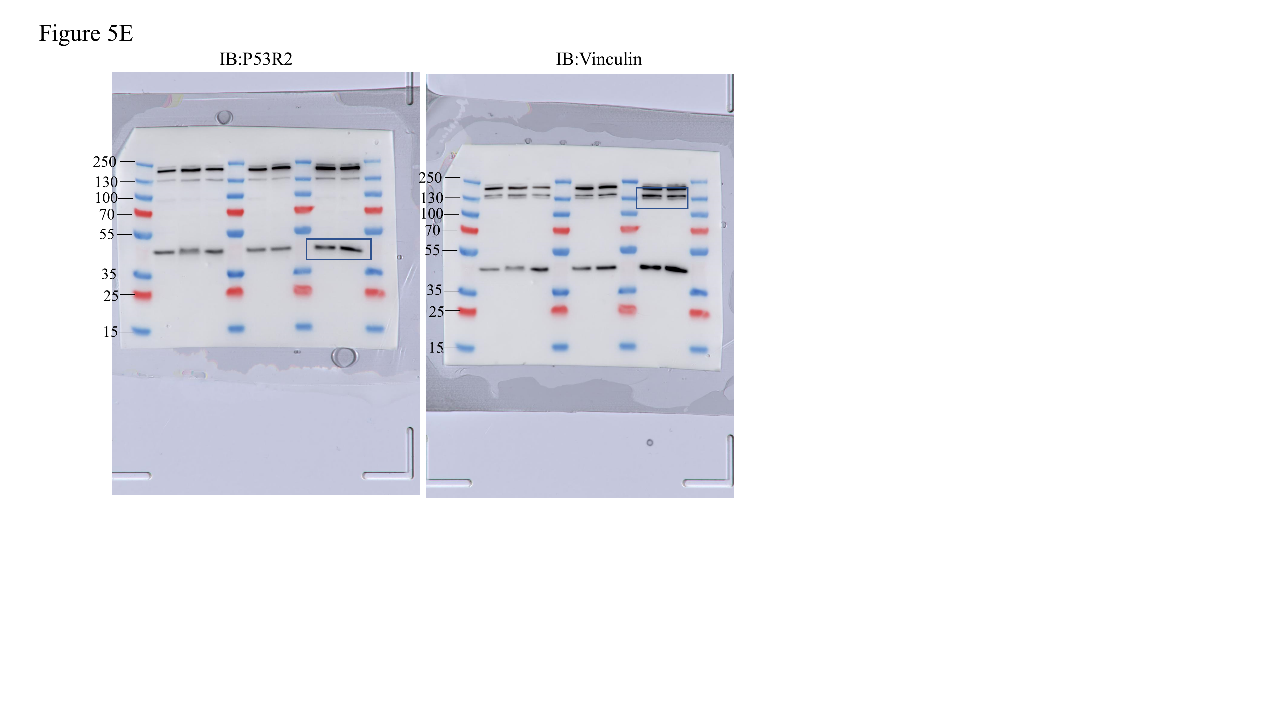


**Uncropped picture for Figure 5E**. The areas marked with boxes are presented in the indicated figures.


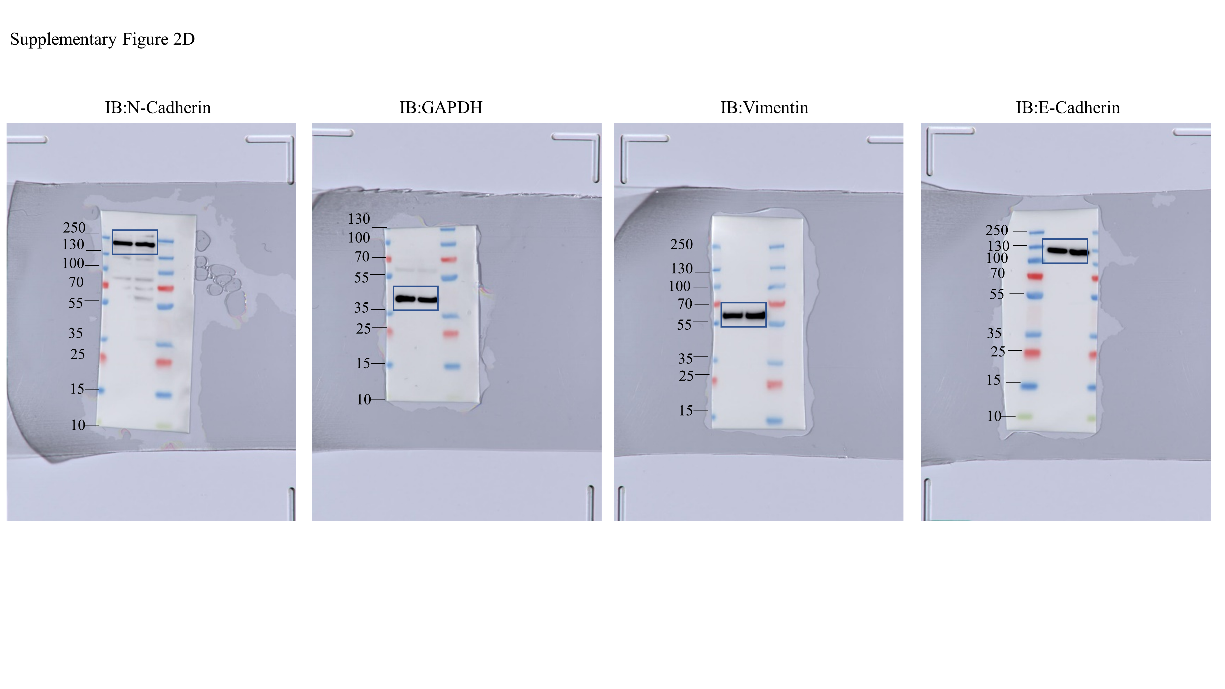


**Uncropped picture for Supplementary Figure 2D**. The areas marked with boxes are presented in the indicated figures.


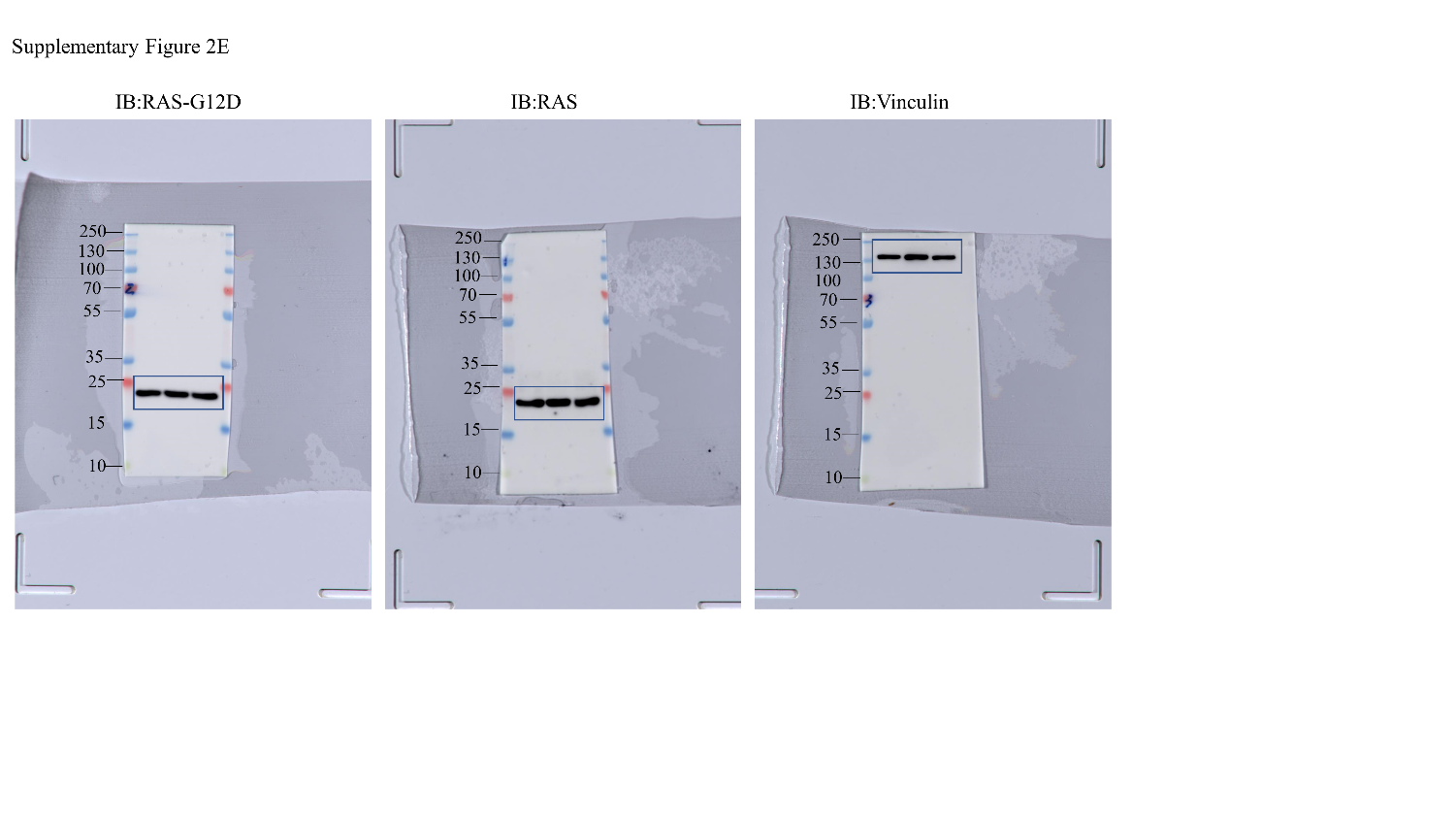


**Uncropped picture for Supplementary Figure 2E**. The areas marked with boxes are presented in the indicated figures.
